# Winter Is Coming: A Southern Hemisphere Perspective of the Environmental Drivers of SARS-CoV-2 and the Potential Seasonality of COVID-19

**DOI:** 10.3390/ijerph17165634

**Published:** 2020-08-05

**Authors:** Albertus J. Smit, Jennifer M. Fitchett, Francois A. Engelbrecht, Robert J. Scholes, Godfrey Dzhivhuho, Neville A. Sweijd

**Affiliations:** 1 Department of Biodiversity and Conservation Biology, University of the Western Cape, Cape Town 7535, South Africa; 2 Elwandle Coastal Node, South African Environmental Observation Network (SAEON), Port Elizabeth 6031, South Africa; 3 School of Geography, Archaeology and Environmental Studies, University of the Witwatersrand, Johannesburg 2050, South Africa; Jennifer.Fitchett@wits.ac.za; 4 Global Change Institute, University of the Witwatersrand, Johannesburg 2050, South Africa; Francois.Engelbrecht@wits.ac.za (F.A.E.); Bob.Scholes@wits.ac.za (R.J.S.); 5 Department of Microbiology, Immunology and Cancer Biology, Myles H. Thaler Center for AIDS and Human Retrovirus Research, University of Virginia, Charlottesville, VA 22903, USA; godfreydzhivhuho@gmail.com; 6 Alliance for Collaboration on Climate and Earth Systems Science (ACCESS), Council for Scientific and Industrial Research (CSIR), Pretoria 0001, South Africa; nsweijd@access.ac.za

**Keywords:** COVID-19, environmental influences, humidity, SARS-CoV-2, seasonality, temperature

## Abstract

SARS-CoV-2 virus infections in humans were first reported in December 2019, the boreal winter. The resulting COVID-19 pandemic was declared by the WHO in March 2020. By July 2020, COVID-19 was present in 213 countries and territories, with over 12 million confirmed cases and over half a million attributed deaths. Knowledge of other viral respiratory diseases suggests that the transmission of SARS-CoV-2 could be modulated by seasonally varying environmental factors such as temperature and humidity. Many studies on the environmental sensitivity of COVID-19 are appearing online, and some have been published in peer-reviewed journals. Initially, these studies raised the hypothesis that climatic conditions would subdue the viral transmission rate in places entering the boreal summer, and that southern hemisphere countries would experience enhanced disease spread. For the latter, the COVID-19 peak would coincide with the peak of the influenza season, increasing misdiagnosis and placing an additional burden on health systems. In this review, we assess the evidence that environmental drivers are a significant factor in the trajectory of the COVID-19 pandemic, globally and regionally. We critically assessed 42 peer-reviewed and 80 preprint publications that met qualifying criteria. Since the disease has been prevalent for only half a year in the northern, and one-quarter of a year in the southern hemisphere, datasets capturing a full seasonal cycle in one locality are not yet available. Analyses based on space-for-time substitutions, i.e., using data from climatically distinct locations as a surrogate for seasonal progression, have been inconclusive. The reported studies present a strong northern bias. Socio-economic conditions peculiar to the ‘Global South’ have been omitted as confounding variables, thereby weakening evidence of environmental signals. We explore why research to date has failed to show convincing evidence for environmental modulation of COVID-19, and discuss directions for future research. We conclude that the evidence thus far suggests a weak modulation effect, currently overwhelmed by the scale and rate of the spread of COVID-19. Seasonally modulated transmission, if it exists, will be more evident in 2021 and subsequent years.

## 1. Introduction

**Figure 1:**
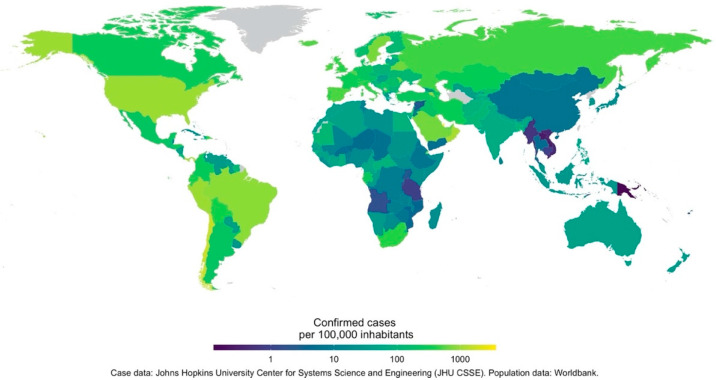
The number of confirmed COVID-19 cases as of 12 July 2020. Data are shown as the number of cases per 100,000 individuals. COVID-19 case data are from Johns Hopkins University Center for Systems Science and Engineering. The world population data are from the World Bank.

A novel coronavirus, thought to have made a zoonotic transition from bats, infected a human host in Wuhan, Hubei Province, China [1]. By late January 2020, the virus, newly named SARS-CoV-2, and the disease it causes, COVID-19, had spread to 18 other Chinese provinces, and to Japan, South Korea, Taiwan, Thailand, and the USA. On the date of submission of this review (15 July 2020), there were 13,331,879 confirmed cases, in virtually every country worldwide (213 countries and territories, Figure 1). At the time, it was reported that 577,825 people infected with the virus had died; both numbers have subsequently risen. The only comparable acute respiratory disease pandemic was Spanish Influenza (H1N1), transmitted from birds to people in 1918, which lasted until 1919 and killed an estimated 50 million people worldwide. In the current highly interconnected world, the impact of the COVID-19 pandemic is likely to be felt for many years [2,3,4]. It is therefore crucial that all potential determinants of the rate and location of the pandemic spread receive careful consideration in order to make appropriate plans for its management.

Epidemiological models have been used worldwide to guide the imposition (or not) of policy and regulatory intervention [5,6]. These models can be modified to include aspects of social characteristics of the infected populations; and they can also be adapted to reflect the modulating effect of environmental influences on the processes that determine transmission. 

Many related respiratory diseases show a connection between climate variables and the dynamics of the disease. It is thus plausible that such a dependency could exist for SARS-CoV-2 (reviewed in Section 4). Given that the COVID-19 outbreak began in mid-winter in the northern hemisphere, where it was (at the time of writing) peaking toward the middle of the boreal summer, and that the opposite scenario seems to be playing out in many southern hemisphere countries, it is tempting to associate this pattern with climate seasonality, as many publications have suggested. However, it is also plausible that the association is spurious, related simply to coincidental spatial connectivity between countries. It is necessary to critically assess the evidence for environmental sensitivity, in both the virus and the disease, before arriving at conclusions that may have significant implications. 

In terms of a response to the pandemic, we need to understand whether and how environmental variables influence the infection rate. This knowledge provides clues for policy and practice to reduce the spread of the virus and potential for treatment options. For example, if analyses show that absolute humidity is strongly associated with reduced infection rates (e.g., influenza [7,8]), artificially raising indoor absolute humidity during periods of low ambient humidity may be an effective intervention. 

Second, if environmental variables do influence the trajectory of the pandemic, the seasonal progression of the disease will lead to different implications across the globe, varying by hemisphere, region, and climatic zone. In the extratropical northern hemisphere, there would be a real possibility of a second wave appearing during the next winter [9]. Conversely, there is a danger that the initially slow pace of the epidemic in the southern hemisphere could be misinterpreted to mean that proactive management has supressed the disease spread. Given that in the south, where the peak of COVID-19 incidence is likely to coincide with the winter peak of other endemic respiratory illnesses, complicating diagnosis and placing additional strain on the health systems, missing the environmental drivers of COVID-19, if they exist, would be profoundly damaging. As we will argue, many southern hemisphere countries are particularly vulnerable (they are in the developmental ‘Global South’ as well as the geographical south). For these regions especially, clarifying the environmental sensitivity will assist the prioritisation of resources.

Third, for longer-term management of the disease, we need to understand whether the seasonal effect will manifest as it does in established or endemic respiratory viruses, in the absence of being able to predict in what period of time (in years) the virus will be eliminated [10].

In this review, we consider all the pertinent studies relating to the effect of a range of specific environmental and climatological variables on the biology of the virus and the epidemiology of the disease.

In Section 2, we develop our reasoning for why southern hemisphere countries can benefit from the lessons learnt in the north, if the application of that knowledge takes heed of particularly southern hemisphere issues. In Section 3, we briefly present the main classes of epidemiological models, since key parameters revealing environmental modulation are derived from such analyses. In Section 4, we explore environmental sensitivity in extant respiratory viral diseases and past epidemics in order to suggest why seasonally coupled environmental influences are also *likely* to exist for SARS-CoV-2. Section 5 then critically reviews evidence for such signals in the literature that had accumulated to 15 July 2020. Section 6 summarises our findings, and offers suggestions for future analyses of the seasonal modulation of COVID-19.

## 2. Why the Southern Hemisphere Is Different

The situation regarding COVID-19 in southern hemisphere is different from that in the north in three ways. First, while the northern hemisphere is moving out of winter at the time of their peak of infections, the southern hemisphere is moving into winter. Second, a much larger proportion of countries in the southern hemisphere are developing countries, with significant resource limitations in their healthcare systems. Third, many of the countries in the southern hemisphere, and on the African continent in particular, have a much higher incidence of pulmonary diseases such as tuberculosis, immunocompromising diseases such as HIV-AIDS, and a higher prevalence of diseases such as cholera and malaria, which may not be recognised as comorbidity risks in COVID-19 but do place coinciding stressors on the health system. To their advantage, the delayed arrival of COVID-19 in much of the southern hemisphere has allowed these countries the time to observe the efficacy of containment and treatment practices in the Global North, and to adapt their healthcare and policy response accordingly. 

The initial outbreak of COVID-19 in China, early epidemics in Iran, Italy, and later much of Europe and the United States took place during the coldest months of their year, and were distributed within a narrow climatic band [11,12]. During the early period of the outbreak in January and February 2020, few known cases had been recorded in the southern hemisphere, which was experiencing peak summer conditions. This could reflect a climate sensitivity, but could just as plausibly reflect dominant trade and human movement patterns [13]. Thus, the initial relatively low rates of spread and mortality in southern Africa, Australia and some regions of South America may simply be a result of being at an earlier stage in these epidemics. However, in both the northern and southern hemisphere, influenza and other coronavirus diseases peak during their respective winter seasons [14]. Thus, if climate factors do play a role in COVID-19 infection rates, the concurrence of transition of southern hemisphere countries to their winter season with the mid-stages of the disease transmission trajectory is of concern, especially with respect to containment policy and health system resource allocation.

The status of healthcare services in the Global South is of concern even without a climatic component to COVID-19. While Australia and New Zealand have healthcare services as good as any in the northern hemisphere [13], much of South America and sub-Saharan Africa struggle with access to quality healthcare. This is associated with poverty and socio-economic inequalities and result in poor health outcomes and financial risk to the state and individuals [15,16,17,18]. The healthcare sectors are understaffed, underresourced, and understocked under normal conditions, which were working at maximum capacity even before the COVID-19 pandemic [19], and will be severely challenged as COVID-19 cases increase [20,21]. Early evidence from China shows a significant correlation between mortality and the healthcare burden in COVID-19 cases [22]. Efforts to model the preparedness of African countries have highlighted concerns relating to the staffing of testing centres, stock for testing, and the ability to implement effective quarantining both inside and outside of healthcare facilities [20]. The prevalence of pre-existing infectious diseases compounds this issue. In the period 2016–2018, 41 African countries have experienced at least one epidemic, while 21 have experienced at least one epidemic per year [23]. South America is currently struggling with outbreaks of measles in 14 countries, and a tripling of the incidence of Dengue Fever in four countries [24]. Recent outbreaks of diphtheria, Zika and Chikungunya have further stretched the healthcare systems [24]. The most prevalent infectious diseases in sub-Saharan Africa include cholera, malaria, viral haemorrhagic fever, measles and malaria [19]. 

Of particular concern in the Global South is the possibility of comorbidity with HIV-AIDS and tuberculosis (TB). Many TB cases are pulmonary in nature [25], while patients with HIV are significantly immunocompromised [26]. There is considerable TB-HIV comorbidity [27]. Corbett et al. [28] found a 38% incidence of HIV in TB-infected patients across Africa, and for the countries with the highest HIV prevalence, up to 75% of TB patients also tested positive for HIV. Comorbidity has decreased from 33% to 31.8% over the past decade, and over the period 1990–2017, TB incidence, TB mortality rate and HIV-associated TB have declined in a number of southern African countries [26]. South America has much lower cases of both HIV and TB, and a comorbidity of approximately 10% [29]. While results from Spain suggest that HIV-positive patients currently on antiretroviral treatment have no higher risk of severe SARS-COV-2-induced illness [30], the comorbidity of those with a longer HIV history and TB comorbidity, with or without HIV, is unknown. There are further related concerns pertaining to continued HIV [31] and TB [32] care during COVID-19, as social distancing requires people to stay indoors and hospitals are overstretched. 

Finally, the relatively delayed spread of COVID-19 to the southern hemisphere has allowed these countries to ‘get ahead of the curve’ through evidence-based management derived in the north [33]. Recent experiences of two Ebola epidemics have meant that many countries in sub-Saharan Africa implemented temperature screening at airports long before the first COVID-19 cases were reported [20], and contact tracing and epidemic management plans are in place [19]. South Africa, Kenya, Uganda and Zambia were reported as having all been particularly proactive in planning for their eventual COVID-19 cases [19]. South America has arguably not been as prepared (Rodriguez-Morales et al. 2020). Studies on modelling risk for the African continent are largely related to importation risk [20], which has been capped due to lockdown in many countries. This form of response is important in delaying the peak and “flattening the curve”, but is unlikely to completely avoid extreme pressure on already stressed healthcare systems [16,22].

## 3. Monitoring and Modelling the Spread of COVID-19

### 3.1. Data Issues

When assembling datasets from many different locations to test the effects of environment on COVID-19 progression, it is essential that the criteria for determining the infection and mortality rates are consistent across sources. The data used to calibrate and validate epidemiological models (e.g., the COVID-19 Data Repository, Center for Systems Science and Engineering (CSSE), Johns Hopkins University) consist of time series of infections, which often include only those with symptoms sufficiently severe that the patients sought medical assistance, and who subsequently tested positive using a PCR-based test for the presence of the SARS-CoV-2 virus [34]. This is known as the ‘case rate’. As the number of tests increases and includes community-based testing, as opposed to testing only those displaying symptoms, the case rate will converge on a true infection rate. PCR testing is accurate (though reporting is often delayed by days to weeks [35]), but if testing is mostly performed on those presenting symptoms and their close contacts, estimates of the true infection rate inevitably include large biases, especially given the high occurrence of asymptomatic or mild cases. Compensating for this bias requires that the sample frame be weighted to be representative of the population as a whole. As antibody-based tests become more widely used, datasets that indicate post facto what fraction of the general population was exposed to the virus will emerge. Antibody tests have variable accuracy, both in terms of false positives and false negatives [35]; nevertheless, their overall accuracy is much better than the guesswork that otherwise goes into estimating the number infected from the medical case rate alone. It is suspected that mildly infected people and even asymptomatic cases can spread the disease [36], but perhaps less effectively than severely ill individuals. It is likely that recovery from SARS-CoV-2 provides subsequent immunity, with initial indications that this may be persitent [37].

The models that predict mortality use a time series of recorded deaths. At a minimum, this includes the deaths recorded in hospitals for people being treated for SARS-CoV-2 at the time of death. More complete records are supplemented with data on people who died in the community or in nursing homes, and were inferred from the symptoms they displayed to have died from COVID-19. For severely-affected areas, it is possible to estimate the anomalies between the COVID-attributed death rate relative to the seasonally adjusted expected population death rate, and infer that these additional deaths (‘excess deaths’) were caused by the pandemic [38]. Where this has been carried out, it suggests that the death rate is substantially higher than that initially reported; however, this approach conflates deaths directly caused by SARS-CoV-2, and those that may have resulted from overburdening the health system. 

Making accurate estimates of transmission rates requires a sufficient number of cases. Often the models are initiated only once 30 or 100 cases have occurred in a location [39] so that the effect of importation of cases due to travelling may be minimised. Therefore, if the area selected for analysis is too small, the number of cases may be inadequate to support the more data-intensive approaches. In most countries, data are collected daily, but the daily data show a lot of noise, partly for stochastic reasons; also, for spurious reasons such as the effect of weekends, laboratory delays, or recoding the date of reporting rather than the date of testing or infection (Section 5.1). Smoothing the data over periods of a week helps to solve irregular daily data patterns [40,41], but this also means that the analyses are unresponsive to events at finer timescales.

The need to match the time period for which infections and deaths are recorded and the period over which environmental drivers are integrated is widely accepted. Similar considerations also apply to spatial resolution. COVID-19 outbreaks are apparently highly clustered, often in small areas. Environmental drivers are also spatially heterogeneous, some much more so than others. The resolution chosen for the environmental data needs to be appropriate for both the grain of the infection process and the grain of the environmental variable. 

### 3.2. Epidemiological Models of COVID-19

Several analytical typologies have been applied to epidemiological models, mostly based on what factors they take into account [42]. Table 1 is a pragmatic classification of the types thus far predominantly used for COVID-19 projections, based on the logic of their construction. Most of these model types can be implemented either deterministically or stochastically for age-structured or non-age-structured populations; for a single, equally-exposed population or for a spatially disaggregated population with transfers between groups; and using frequentist or Bayesian approaches.

Simple extrapolation and phenomenological models are suitable for projections of less than one month into the future, whereas the somewhat mechanistic epidemiology models are more robust for projections months or years into the future. The various classes of models can in principle run at any spatial scale and over any time period, but in practice there are data-imposed constraints. 

### 3.3. Incorporating Environmental Drivers into Epidemiological Models

**Figure 2:**
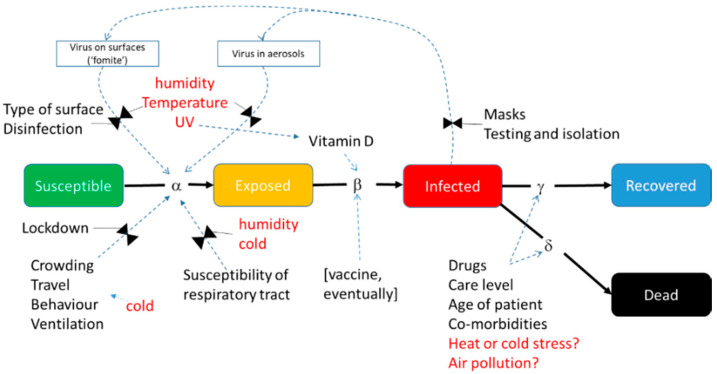
Environmental factors that have been suggested to influence a COVID-19-like disease, overlain on the structure of a generic SEIR-type compartment model to show the potential mechanisms of action.

Environmental influences can be introduced into the basic model structures at a variety of points (Figure 2). Where they are introduced and what the models are able to say about the relationship between the environmental influences and infection or mortality rates depend on the theoretical basis of the model (Table 1). Models that best capture the functional relationship of confirmed daily cases across time are best suited for revealing environmental drivers. The phenomenological and compartmental models are the strongest contenders here. The raw time series of confirmed infections and deaths can be time aggregated, and time lagged with respect to the environmental factors, to find the best fits, as long as this is performed consistently, and considers the time lags already built into the model structure.

One approach is to establish correlations, either over time or across space, between the infection rate at a given time and simultaneous metrics of environmental factors such as temperature, humidity and UV (see Section 5.4). In SEIR and similar models, two metrics are available for this infection rate: *R*_0_, the Basic Reproductive Number, and *Rt*, the Effective Reproductive Number. *R*_0_ is defined as the expected number of secondary infectious cases generated by any single average infectious case in an *entirely susceptible* population. *R*_0_ should be largely free from signals attributed to imposed factors that affect human behaviour. It is typically derived from the initial portion of the growth curve when the disease spreads in a population where everyone is susceptible, before control measures have been put in place (i.e., completely ‘natural conditions’ sensu Shi et al. [50]) or herd immunity had been attained. Neher et al. [51] note that “*R*_0_ is not a biological constant for a pathogen” (p. 1) but it is affected by factors such as the infectiousness of the virus, susceptibility of the hosts (e.g., due to age or an assortment of comorbidities), duration of the infectious period, density of susceptible people (also population density and the proportion of the population that is urbanised) or the contact rate with them (including aspects of mobility), and environmental influences (as shown in Figure 2). These aspects are subject to localised idiosyncrasies across the globe and must be accounted for in regional or global analyses when calculating or comparing *R*_0_.

*R_t_* is a measure of observed disease transmissibility, defined as the average number of people a case infects at any time (*t*) once the epidemic is underway. *R_t_* incorporates changes in a society’s behaviour (self-regulated responses and non-pharmaceutical interventions or NPIs [52]) as the disease becomes widespread, and varies day to day. These effects are typically stronger than the environmental influences, and can easily mask them or generate spurious associations. It is not advised to base assessments of environmental effects on *R_t_* due to the ‘noise’ that the signal will contain, unless there is sufficient information that permits inclusion of the interventions as continuous, time-varying factors. 

For the compartment models, it is possible to derive the values of the key model parameters by model inversion, in near-real time, and from these, calculate *R*_0_. This needs at least one more observation than there are free parameters to be estimated. In practice, accurate estimates of confidence limits require many more data points than parameters. The multiple observations can come from a single-population time series, but this would limit the degree to which changes over time can be resolved within the parameters themselves. If there are multiple time series from different populations, both temporal and spatial variation of the parameters can be obtained. 

Phenomenological approaches typically use a variety of parametric regression models (see Section 5.4). It is sometimes necessary to fit a piecewise model to accommodate the breakpoint that develops when country-specific NPIs are introduced. It is generally only possible to compare the parameters of the curves across locations (rather than within locations, over time) to determine whether there is a systematic pattern that relates to any environmental predictors. This is because fitting multi-parameter non-linear curves using data from only a part of the curve (in epidemics, usually just the initial part) is notoriously difficult and uncertain. If the effect of the environmental factors on the model parameters was known, they could be used to alter the curve parameters dynamically, and thus the projected outcomes; but the parameters typically have no intrinsic biological meaning.

## 4. Implications for COVID-19 of Environmental Sensitivity in Other Viral Respiratory Diseases 

Seasonality of prevalence is a common feature in most persistent and established or endemic respiratory infectious disease [53,54,55,56,57,58,59,60], as well as many other infectious diseases [61,62], in diseases (or endemic tolerated infections) of both humans and other animals. Peaks incidence periods occur during the shoulder seasons or the winter, oscillating globally with the opposing boreal and austral climate. Seasonally varying prevalence has a general latitudinal gradient and is accentuated in highly seasonal temperate and subtropical climates (with some rare exceptions) but is also observed in tropical regions [63]. Seasonality is found in a wide range of viral respiratory diseases (VRDs)—including influenza viruses, para-influenza virus (PIV), human syncytial virus (RSV), rhinoviruses and human coronavirus strains (HCoV) [55,60]. For endemic viruses causing VRDs in humans, seasonal peaks are usually quite predictable, but interannual variability in onset and duration of any season, and the virulence of respective seasonal strains, vary. It follows, therefore, that if VRD prevalence follows this climatological pattern, a mechanism(s) that connects and modulates the viral disease progression with seasonally varying climatological variables in individuals or populations must exist. This sensitivity must occur in at least one location of the SEIR model (Figure 2). 

In the case of novel viruses, the role of seasonality is more contentious, mainly because they have not existed for enough time for seasonality to be unambiguously established. The seasonal prevalence of pandemic strains of virus is often conflated with the so-called second wave, which may be coincidentally associated with the following winter season, suggesting that there is a climate-based modulating effect on its incidence [10,64]. In the case of SARS and MERS, the attribution of resurgence to climatological drivers, as opposed to secondary circulation dynamics, remains unresolved [65,66]. Novel viruses are much less predictable than established viruses with respect to their persistence, re-emergence in the following years or seasons, and virulence in later outbreaks [64,67]. Until a novel virus becomes endemic and recycles (in its existing form or as mutated strains), its seasonal prevalence is difficult to assess [68]. The magnitude of the current SARS-CoV-2 pandemic is likely to result in an extended period of persistence [69], and thus if seasonal prevalence exists, it should eventually be unambiguously apparent.

In the generalised SEIR model shown in Figure 2, environmental modulation can primarily take place at two stages, namely Susceptibility and Exposure. Environmental sensitivity insights can come from two basic sources. The first is observational data and laboratory studies and analyses of the environmental modulation on the SARS-CoV-2 virus biology and the incidence of the disease it causes (as in this review). Second, we can examine data and information from published studies on respiratory viruses and VRDs and related endemic and novel coronaviruses specifically (see [53,55,57,59,60,67,70] for general treatment of this topic). 

In this section, we examine three sets of hypothetical mechanisms which explain environmental modulation and seasonality of VRDs other than COVID-19: (i) physical environmental variable modulation, (iii) biological and host behavioural modulation, and (iii) viral molecular and biochemical modulation.

Physical environmental variable modulation hypotheses focus on the meteorological correlates of seasonality in the diseases [54,58] and comprise the bulk of such studies. These all follow the basic tenet that selected environmental variables (such as temperature or humidity) vary in space and time with the progressing seasons, and if a mechanism that links them with a VRD can be demonstrated, this makes them a suitable candidate for explaining VRD seasonality. There is a lack of clarity in the literature regarding which definition of humidity is best applied as environmental moderator of respiratory viral epidemiology. Studies employ relative humidity (RH), absolute humidity (AH), specific humidity (SH), vapour pressure or dew point (more in Section 5 below). This renders comparisons and conclusions difficult to reach [7]. RH and SH have strong dependence on temperature, which further complicates studies that include both temperature and humidity as predictors.

The postulated mechanisms are usually tested in laboratory studies which monitor the persistence of viable viruses in aerosol droplets and on surfaces [71], perform experimental transmission studies in animal models [72], or study the relationship between observed ambient or indoor environmental variability and infection rate, morbidity and mortality, with the assumption of causality (Section 5). Notably, results from temperate and tropical climate zones (or with ranging latitude) are often contradictory. This has led to a suggestion that different seasonality mechanisms are at play in different climate zones: humidity (aerosol droplet transmission) as the key driver in temperate regions, and precipitation driving behaviorally mediated contact transfections in the tropics [73,74,75]. 

The environmental determinants of virus transmission in aerosol liquid droplets have received substantial consideration. The premise is that, in winter, characterised by relatively lower humidity, pathogen-bearing aerosol droplets (PBADs) are more persistent. PBADs expelled by infected individuals often contain viruses or bacteria, in a mixture of mucus, saliva and dissolved salts, and can travel up 8 m from a simple sneeze [76]. Upon leaving the airway with moisture saturation close to 100%, PBADs are exposed to much drier air which results in evaporation. They can quickly lose up to 90% of their water mass and reduce in size. At an RH of 40–60%, the water loss greatly increases the salt concentration to levels that inactivate viruses. In contrast, for RH < 40%, the dissolved salts precipitate, resulting in a PBAD with low salt concentration and a high number of infectious viruses [77]. PBADs range in diameters 5–20 μm when the ambient RH is 30–60%, whereas below 30%, a PBAD may immediately reduce its size below 0.5 μm, and become a droplet nucleus [78,79]. Thus, conditions of lower ambient RH result in the production of smaller, lighter (longer floating periods), and potentially more penetrative PBADs, thereby elevating the exposure component of the SEIR model [80,81]. 

The role of temperature in influencing the prevalence of VRDs is more contested and complex. This is partly because temperature and AH together determine RH, which affects the rate of evaporation and thus PBAD dynamics, as argued above [72,82]; and temperature could also have direct effects. Several studies associate temperature with respiratory disease incidence, some by direct association [83,84] and some focussed on the temperature changes (i.e., lowering temperatures rather than lower temperatures [85]). Temperature may also play a mediating role in other ways. The first set of hypotheses consider the direct effect of temperature on respiratory virus survival. There are very few such studies but they show that viruses in general are surprisingly tenacious, with survival periods of days at room temperature for SARS-CoV-1. Effective inactivation occurs at temperatures of above 56 °C [86,87].

Another temperature-mediated mechanism with substantial literature involves the fomite viability of viruses [88,89], particularly in public spaces and hospitals, involving endemic coronaviruses and SARS-CoV-2 [90,91]. Some studies explore the role of temperature alone on specific surface types [88], while others look at the combined role of temperature and humidity [90,92]. Respiratory viruses, including human coronaviruses, can remain viable as fomites on a range of surface types, indoors and in sheltered external environments, at room temperature and higher, for periods of hours to days and from days to weeks on refrigerated surfaces at 4 °C. Persistence depends on both the surface type and the temperature and humidity range (see Table 1 [91] for a recent summary). Thus, the risk of infection from fomites (the exposure element of the SEIR model) increases as temperature decreases. The combination of temperature and humidity has been found important for fomite viability in the endemic human coronavirus HCoV 229E (Table 2). Most studies aim to test sterilisation techniques and the efficacy of personal protective gear [91,93,94,95,96]. One hypothesis posits a predominance of surface contact transmission in the tropical climates, versus transmission through PBADs in temperate climates [97]. 

A range of other physical environmental variables have been cited as moderators of respiratory viral epidemiology. They often co-vary with other causal variables. Wind and wind speed are relatively neglected as physical environmental factors in infectious disease epidemiology. Given that windy seasons occur in many climates zones, wind should not be discarded as a contributing variable [98]. For influenza, wind has been cited in some instances as a factor in transmission of infectious particles from remote locations, as promoting the extended local transmission of PBADs [99,100], with a convincing account in one case of equine influenza [100]. Barometric pressure has also been considered, for example in the case of respiratory syncytial virus, where it was found to have no statistically significant influence [101]. In other studies, it does have an influence, along with temperature [102]. Guo et al. [103] found air pressure to be a predictor of the risk of influenza infection in children in Guangzhou, China, with a differential effect by age.

Rainfall seasonality and disease incidence in general are well described [104], but literature on the relationship between rainfall patterns and VRD epidemiology is restricted to tropical climates. Most studies have considered rainfall either at a very local scale, or as part of a set of meteorological variables being tested. Pica and Bouvier [105] comprehensively review the literature on this rainfall and VRDs, and conclude that for a range of respiratory viruses (primarily influenza and RSV), there are as many studies finding some association as there are studies finding no link. With attenuated intraseasonal temperature variation in the tropics, rainfall provides a key differentiator between seasons, possibly explaining the strong associations between rainfall and respiratory illness prevalence there. The mechanism of association is less clear. There is a suggestion that the tropical rainy season causes crowding, and thus increased exposure [106], another suggesting that reduced sunlight is associated with pneumonia incidence [107], and yet another citing diurnal temperature changes [108]. The improvement in air quality and reduction in allergen production following rainfall may be another mechanism [109].

Solar ultraviolet radiation (solar UV) varies greatly with season everywhere and is thus an attractive candidate to explain seasonality of VRDs. UV radiation in laboratory settings is a very effective means of deactivating viruses, and there are a plethora of studies of this effect on all kinds of pathogens (including coronaviruses SARS-CoV-1 and MERS-CoV), mainly targeting hygiene and outbreak management in public spaces and hospitals [110,111,112]. Studies that consider the environmental effect of solar UV (a component of sunlight) without confounding effects of other variables are rare. Sagripanti and Lytle [113] state that, for influenza, “the correlation between low and high solar virucidal radiation and high and low disease prevalence, respectively, suggest that inactivation of viruses in the environment by solar UV radiation plays a role in the seasonal occurrence of influenza pandemics” but concede that there are a range of additional factors that need to be considered. Despite UV being regarded by several authors as the “primary germicide in the environment”, its independent effect as a seasonal driver of VRDs remains uncertain (on this point, for influenza, see [114]).

A second set of hypotheses for explaining the seasonality of VRDs consider behavioural and physiological responses to changing environmental variables such as temperature [54,55,60], related mostly to the exposure but also the susceptibility component of the model in Figure 2. These include considering the consequences of confining people in sheltered and enclosed spaces during colder weather, with recirculating air and closer proximity to infected co-inhabitants, thus increasing the likelihood of exposure. They also include the idea that exposure to colder and drier air at the cellular level in the respiratory tract results in impaired physical or immune-system defences to infection, and hence increased susceptibility [60,115]. Large (<30 μm) and medium (<10 μm) inhaled PBADs are normally captured in the upper nasal mucosa and upper respiratory tract, respectively, and are transported towards the mouth (and expelled) through a synchronised circular movement of cilia. The combination of the mucosal layer and cilia can effectively clear the particles [79]. However, low ambient RH has been demonstrated to reduce the effectiveness of both mucosal production and cilia action [60,72]. A corroborating study demonstrates that dry air (low RH) impairs host defence against influenza infection in genetically engineered mice with human-like lung tissue, as well as slowing recovery [116]. 

The third set of hypotheses consider the biochemistry and molecular adaptation of the viral pathogens [55]. These take into account the temperature sensitivities of the various stages of the virus infection cycle, from binding to the host cell, replication of nucleic acids, the stability of secondary structures of viral proteins, and eventual ejection of the virus from the host cell [55]. Given that there is a gradient of temperature within the respiratory tract, and that breathed air can greatly alter conditions in the upper respiratory tract, susceptibility can increase under cold conditions, especially to viruses which are adapted to be most efficiently infectious at temperatures slightly below normal body temperature [55,115]. 

Falling somewhere between the physical, physiological and biochemical hypotheses in explaining seasonality of respiratory viruses is the change in susceptibility with varying serological levels of vitamin D. Vitamin D synthesis occurs when the skin is exposed to sunshine, which varies seasonally (confounded with UV, temperature and other variables). Vitamin D has been suggested as an important form of defence against microbes, influenza and pneumonia in particular [117,118,119,120]. Shaman et al. [121] attempted to model this effect on influenza prevalence in the USA and concluded that seasonal variability in other factors such as humidity and even the school calendar were better at explaining their results. 

These considerations are incomplete, with a final abiotic aspect that must be included. Air pollution refers to a wide range of harmful, primarily geogenic (naturally occurring) and anthropogenic particulate matter, chemicals or gasses that cause negative or dangerous physiological responses and effects in humans and biota. It is well known that poor air quality can have direct and indirect impacts on human health, and in particular on the susceptibility of humans to respiratory viral infections as well and a measurable effect on the severity and mortality rates [122]. Gases such as nitrogen dioxide, ozone and especially particulates classified by size (PM10, PM2.5, and PM0.1) have different pathological mechanisms and effects but are all known to be associated with the increases in viral respiratory disease incidence, hospitalisation or attributed deaths, famously during the London fog of 1952 [123] and the 1918 Spanish Influenza Pandemic [124]. Clifford et al. [125], for example, showed that PM10 inhalation exacerbates the response to influenza, and Ye et al. [126] showed that ‘haze’ (a combination of air pollutants) was associated with the spread of respiratory syncytial virus in children. Air pollution is also known to have a strong seasonality, driven by both seasonal economic production activity and also by ranging seasonal metrological conditions which can either concentrate and trap pollutants in surface air or conversely disperse pollutants and improve air quality [127,128,129]. Therefore, it is a further consideration that seasonal variation in air quality and pollution is an additional factor for consideration as a contributor to the seasonality of respiratory viral infections that have been reported.

It is most likely that each of these hypothesised mechanisms has some role, either in unison, or independently or that one mechanism dominates in particular conditions [60]. While the precise mechanism that explains the relationship between environmental factors and disease prevalence is important, particularly because it may reveal optimal management interventions (of transmission and for treatment), statistical attribution of a strong correlate may suffice for effective management [8]. 

## 5. Critical Assessment of Studies of COVID-19 Climate Susceptibility

Evidence from the many studies on viruses not dissimilar from SARS-CoV-2 suggests that a seasonal and environmentally-mediated signal should be seen in the novel COVID-19 epidemic. What do studies to date tell us?

We comprehensively reviewed the preprint and peer-reviewed literature on the topic of environmental influences of SARS-CoV-2 transmission. We used the Boolean search capability of Google Scholar to locate articles with the following keywords in the article title: “(COVID-19 OR SARS-CoV-2) AND (pollution OR humidity OR temperature OR UV OR climate OR weather OR season OR seasonality)”. This returned 287 articles on 8 July 2020. On the same day, additional searches for these search terms were conducted in the title fields on PubMed and the title, abstract and subject fields on the WHO COVID-19 literature database (https://search.bvsalud.org/global-literature-on-novel-coronavirus-2019-ncov/), returning 469 and 170 publications, respectively. All searches were constrained to the year 2020. We selected only those studies on infection rates or similar metrics, excluding studies based solely on mortality rates. The combined set, which contained many duplicates and triplicates due to the intersection of three sets of search results, was screened manually and papers suitable for inclusion in our review were retained. Five reviews in preprint were excluded from our assessment, but we did verify that we included in our analysis all relevant papers cited in these reviews. Since we a priori expected many preprint manuscripts, we did not embark on the review with the intention to be PRISMA compliant (as would be necessary for a meta-analysis and systematic reviews), and hence we did not count the number of duplicates and triplicates, the ineligible studies discarded, or the reasons why they were discarded.

The result of our searches yielded 42 peer-reviewed publications and 80 preprint manuscripts (Supplementary Tables S1 and S2). The peer-reviewed publications were subject to normal review scrutiny, and form the main body of this section. We did not assess the outcomes of the preprint papers (i.e., they are not discussed in detail as part of Section 5.5) in order to avoid erroneous conclusions based on unassessed data, results or interpretations; nor did we attempt to apply our own peer-review process.

The peer-reviewed research conducted on the role of climatic variables in COVID-19 transmission has been highly interdisciplinary, with authors spanning 25 broad academic backgrounds. The largest number of authors (27) currently work in disciplines of geography, earth and environmental sciences, which incorporate climate science. This is closely followed by the 26 authors working in public health, and 25 authors in disciplines of epidemiology, virology and disease control. A total of 40% of the authors are in fields directly relating to COVID-19 and climate. There is, however, a notable spread of authors in more distal academic and medical fields. Notably, the authorship of 18 papers included nobody with an explicitly medical background. Of the multi-authored papers, only three were by researchers who all come from the same disciplinary background, and for two of these, the backgrounds were epidemiology and medical laboratories. 

Collectively, the peer-reviewed studies provide only weak evidence that SARS-CoV-2 is more infectious under lower temperatures and lower levels of absolute humidity. Similarly ambiguous relationships for air pollution, UV and wind are reported, with a smaller focus on these variables in the literature. There are considerable differences in the ways in which the relationships have been established, resulting from which co-varying variables were included or not; use of different metrics of viral transmission, and which statistical methods were applied. In many cases, insufficient information is provided on the methods and data used, making it impossible to replicate the analyses.

### 5.1. Geographical Coverage of Studies

This section is relevant because of the high dependence on spatial variance to provide information at this early stage of the pandemic. The geographical coverage of the literature on the environmental influences on SARS-CoV-2 is heavily weighted to the northern hemisphere. Data from Bolivia, Ecuador, Brazil and Australia were included in only five studies, i.e., one-tenth of the total. Most of the southern hemisphere studies are included in studies claiming to be near global in their sampling. Only eight studies focus specifically on a country in the southern hemisphere, Brazil [130,131,132,133,134,135,136,137], and none of them consider any African country.

### 5.2. Influential Variables

Environmental variables considered in preprint and peer-reviewed publications as modulators of SARS-CoV-2 transmission rates include mean, minimum and/or maximum daily temperature, and diurnal temperature range; an undefined ‘humidity’ variable, relative humidity, specific humidity and absolute humidity; dew point temperature; rainfall; wind speed or wind power; air pressure; some metric of solar or UV radiation; and ‘air quality’ (Supplementary Tables S1 and S2). These choices are apparently strongly influenced by the literature on other viral respiratory diseases.

Which definition of ‘humidity,’ is selected is significant challenge for interpreting and comparing studies. Humidity broadly refers to the amount of water vapour held by air (which effects on the viability of pathogens in exhaled aerosol droplets—see Section 4). Studies must account for the fact that atmospheric pressure and temperature modulate the amount of water that a volume of air is able to hold in a gaseous state. A relatively small amount of water vapour is able to saturate cold air, whereas more water vapour is required to bring warm air to saturation. The studies we reviewed that seek to establish whether humidity is a potential driver of COVID-19 use absolute humidity, relative humidity or specific humidity. Two studies use ‘humidity’ [138,139] without qualifying whether it is relative, specific or absolute humidity. This ambiguous use of the term does not permit reproducibility or meta-analysis. Absolute humidity is defined as the total amount of water vapour held by air, in units of g·m^−3^. A temperature change will not necessarily change the moisture content; it simply changes the capacity of the volume of air to hold water. Only if temperature drops to saturation point, will condensation occur and water vapour content (but not relative humidity) will drop. If temperature increases, water vapour content will only increase if a moisture source is available from where evaporation can take place, or if a moist air mass moves in to replace the drier one. Relative humidity is the fraction of water vapour, expressed as a %, contained by air relative to the amount of water vapour required to result in saturation of air at a given temperature and pressure. Specific humidity is the amount of water vapour per unit mass of dry air (g·g^−1^). The distinction between relative and absolute humidity matters less in situations when the seasonal thermal range is constrained to a narrow band, such as at some mid-latitude coastal locations and near the tropics. However, in space-for-time studies—such as are required for global syntheses of seasonality effects—the reliance on absolute humidity should allow the investigator to arrive at plausible conclusions about atmospheric water vapour’s effect on viral transmissibility [140,141,142].

Environmental data were obtained from various sources such as ERA interim [143] or local meteorological organisations, and maybe provided as daily data or aggregates on temporal scales from 10 days to months. Some use ‘seasonal climatologies’, i.e., averaged long-term data. Since symptoms first manifest 3 to 14 days after infection, analyses sometimes apply lags between the independent and dependent variables of up to 14 [40] or 21 days [41]. Lags have been accommodated in the reviewed literature by applying moving average filters to the daily time series of environmental variables with a width of 7, 14 or 21 days [41]. Another approach is to base the analysis on 10 day aggregates of environmental data [140]. It is uncertain how such discretised intervals can be aligned with case data that is typically daily, but yet contains various delays. Some studies take the mean of the variable over the analytic time period; for example, Jahangiri et al. [144] who ambiguously use either the mean temperature over the study period or over the year, or Liu et al. [141] and Sajadi et al. [12] who use the mean of the environmental variables over the period for which case incidence data were obtained. Most studies, do not account for lag effects [138], or if they do, fail to adequately explain how lags were accommodated [40].

### 5.3. Dependent Variables

Which metric of SARS-CoV-2 transmission to use as dependent variable is critical in addressing the central question, “do environmental variables modulate the transmission of the virus?” We argue in Section 3.3 that the Basic Reproductive Number, *R*_0_, is the best parameter for this purpose since it excludes the effects spontaneous or imposed non-pharmacological control measures implemented to slow the spread of the disease, but which still incorporates the environmental influence of a particular place. The failure to adequately account for non-entrée influences is the Achilles’ heel of many of the studies reviewed. Of the literature we assessed (Supplementary Tables S1 and S2), only six studies base their assessment of the presence or magnitude of environmental influences on *R*_0_ as the dependent variable [39,145,146,147,148,149]. 

Jebril [150], Luo et al. [151], Poirier et al. [142], and Wang et al. [152] used *R_t_* (see Section 3.3) as the response variable. Because *R_t_* is very context specific and sensitive to social factors and interventions, using this parameter to assess the presence and size of environmental influences will in most instances have a low signal: noise ratio. The usefulness of *R_t_* is that it demonstrates how effective NPI measures are in controlling an epidemic, and provides information on how regulators must adapt these interventions over time, based on health and economic goals. The non-environmental ‘noise’ can be filtered out, but this requires a great deal of data regarding the nature of the specific interventions applied, movement patterns, precise knowledge about testing and reporting (which is not necessarily constant), and so forth. None of the *R_t_* based studies to date meet these preconditions, and are therefore not able to remove the non-climatic (social) influences from the rapidly fluctuating *R_t_* values.

Another approach that holds merit is to use the growth rate or doubling time estimated from the exponential increase in cases as dependent variable [148,153,154,155,156,157,158,159,160]. Merow and Urban [156] argue that these kinds of metric are robust even if the details of testing and reporting vary from place to place, as long as the detection probabilities at a place remain constant over the estimation period. This argument is equally valid for estimates of *R*_0_.

Another variation to this theme of estimating growth rate-related parameters as an indication of transmissibility is to take rates as time required to progress from the first reported case to 200 cases [161], or to use the cumulative number of cases reached 28 days after the first reported case [162]. However, these approaches effectively fit a linear model to case vs. time data, which does not account for the accelerating rate of increase in number of cases. Lolli et al. [163] use the daily ICU case anomaly, but this of course entirely excludes all but the most severely ill patients and cannot be seen as being representative of disease transmissibility.

Other data-related considerations, particularly in relation to studies that use parameter estimates of the exponential relationship that daily new infections has with time, are that care must be taken to omit both (i) cases that result from the importation of infected individuals from the time series (i.e., new cases must be local transmissions only), and (ii) the case data obtained after the intervention period begins. Requirement (i) can be affected by including only the portion of the time series after a certain minimum number of cases are present, as has been performed by Caspi et al. [154], Merow and Urban [156], and Notari [157]. Requirement (ii) is met by Ficetola and Rubolini [155], Merow and Urban [156], Notari [157], and possibly for Oliveiros et al. [158], although it is uncertain how strictly this was implemented due to their statement that Oliveiros et al. [158] “considered mainly the initial days of the time series” (p. 4). We will comment on the reproducibility of methods in Section 6. Requirement (ii) is implicit in the definition of *R*_0_, but the two requirements constrain the usable data to between ‘not too early’ and ‘not too late’.

The bulk of the studies in Supplementary Tables S1 and S2 used daily new or cumulative confirmed cases as response variables. This practice is not advised for largely the same reasons given for *R_t_*. Such daily data are likely to carry too many other non-climatic signals to be generally used—unless, of course, analyses included a specific set of controls that would be difficult to extend to the global context.

### 5.4. Modelling Approaches

The studies in Supplementary Tables S1 and S2 employed the following statistical methods to evaluate relationships between environmental variables and the transmission rate of SARS-CoV-2: various linear, logistic, or exponential parametric models [39,41,50,130,134,142,146,148,151,158,164,165,166,167], sometimes with the inclusion of non-Gaussian error structures as permitted by Generalised Linear Models (GLMs) [141,157,162,164,168,169,170,171]; Generalised Additive Models (GAMs) [41,134,167,172,173]; distributed lag panel regression models [153]; machine learning such as support vector machines and decision trees [147]; local panel projection estimator within a country-level dynamic framework [174]; Loess smoothers/curves [142]; Bayesian methods [157]; and Pearson’s, Spearman’s, and Kendall’s correlations [40,138,139,140,154,175,176,177].

Regression approaches allow functional relationships to be established between the driver (any of the environmental influences) and response variable (a metric of infection rate), allowing the magnitude of the environmental effect can be determined. Robust implementation of a regression approach would include place as a random effect (i.e., as mixed models, also known as panel regressions; for example, [11,153,155,178,179]). This allows the fact that the effect of the environment on viral transmission varies from place to place, for social and historical reasons. Multiple regression allows the simultaneous evaluation of several predictor variables in terms of the influence they collectively or individually have on the outcome [39,180,181]. It is possible to establish which of the drivers, if any, has the greatest contribution to an effect seen in the outcome variable. For example, Mollalo et al. [180] used multiple regression to evaluate the simultaneous contributions of environmental and socio-economic influences on USA county case counts. If parameterised properly, multiple regression can be used to rule out contributions of potentially confounding and multi-collinear variables.

Loess smoothers and correlation approaches, although useful for a qualitative assessment for the presence of environmental influences, cannot inform us about the relative importance of environmental modulators versus other location-specific or social influences. Similar non-quantitative approaches that only hint at the presence of relationships include the simple visual mapping of the number of infections in relation to climate zones or latitudes [12,150,182,183,184]. These methods can at best raise an hypothesis that requires further testing.

Other approaches worth mentioning include the application of wavelet transforms [185], multivariate analyses [130], and ecological niche models [186,187]. Wavelet analysis, which requires a long time series, provides only a qualitative view of disease dynamics as modulated by weather or climate variables. Ecological niche models are not suited for studies on COVID-19 because disease dynamics are entirely different mechanistically from the principles that govern organisms and ecological systems (as reviewed by Carlson et al. [188]). Multivariate methods are useful for examining environmental variable modulation of COVID-19, since they provide many, if not all, of the benefits of multiple regressions, plus they have other features that confer flexibility and the ability to accommodate a range of data types. They are ideally suited for situations where there are many factors that might contribute simultaneously to the variation of one or many outcome variables. The application of a multivariate approach by Auler et al. [130] uses data on the daily new confirmed cases (see critique above), and for this reason we do not consider the findings of this study further in our review.

Dynamic or mechanistic models (predominantly the compartment models of the SEIR family) are useful tools to explore how seasonality may impact on the evolution of the disease, and provide a way to discern the signature of seasonality in near real-time observational data. Such an investigation recently reported on by Baker et al. [189] concluded that under the high infection rates of COVID-19, within the context of almost the entire population being susceptible at the onset of the disease, seasonality effects on the disease evolution will be limited initially. However it cannot be discounted at later stages, if for instance, the immunity gained by recovered patients is temporary, so that they become susceptible again in subsequent years or if herd immunity is not attained before managed abatement of the epidemic (as we are seeing in some countries experiencing resurgences). A similar study by Neher et al. [51] came to similar conclusions.

### 5.5. Findings

We will now discuss only the findings of those studies that have undergone peer review, have selected appropriate environmental data as influential variables, relied on suitable response variables (such as *R*_0_ or parametric estimates) to estimate the local viral transmission rates in the absence of policy control measures, accounted for potential confounding influences, and applied appropriate statistical models.

The only peer-reviewed paper that fulfils all of these criteria is that by Yao et al. [39], which undertakes an assessment of the effects that temperature, relative humidity, and UV radiation have on the *R*_0_. This study has a relatively narrow geographical focus: it includes 227 Chinese cities. *R*_0_ was calculated from data over the period 10 February to 9 March 2020. The authors assert that these data are for the “expected number of secondary cases generated by an initial infectious individual, in a completely susceptible population” [39] (p. 1). All daily environmental data were spatially matched as closely as possible to the cities they represent. Given the large number of cities, each with its unique climate, this kind of study lends itself to a regression-type analysis if each of the daily observations per environmental variable are averaged over the study period duration before relating them to each locality’s *R*_0_. This study did not find an influence due to any of the environmental variables studied on the rate of SARS-CoV-2 transmission. A weakness of the study was the failure to account in their multiple regression model for any of a large number of city-level confounding influences.

A single published study does not provide robust support for the presence or absence of a climatic influence on SARS-CoV-2 transmission rates. The preprint studies [11,147,152,153,155,156,162,174,190] offer mixed statistical support (none, weak, or strong relationships) for the influence of environmental drivers. Carlton et al. [153] show that that UV radiation affects COVID-19 growth rates, but not temperature or humidity. Merow and Urban [156] offer comparable support for a UV radiation effect. According to Ficetola and Rubolini [155] and Wan et al. [190], COVID-19 transmission is greatest at a temperature of 5 and 6.3 °C, respectively; the former authors further show that transmission peaks at a specific humidity, ~4–6 g·m^−3^ (peaking implying optimum conditions above and below which transmission rates drop off). Similarly, Leung et al. [162] suggest support for the hypothesis that lower temperature and humidity enhance COVID-19 transmission. Similar responses are seen by Lin et al. [165] and Wilson [174] with regards to temperature, but they also suggest an interaction between temperature and relative humidity [165] and temperature and mobility [174] in terms of modulating infection rates. In contrast, Gupta and Gharehgozli [147] show that higher temperatures enhance the spread of the disease; they also show that viral transmission is enhanced under higher concentrations of PM2.5.

## 6. Discussion

This pandemic has rapidly mobilised scientists from diverse disciplines in a possibly unprecedented way. Scientists have helpfully offered insights and analytical methods based on their own disciplines They did so efficiently and swiftly, particularly in those countries most heavily affected by the pandemic early on. The rush to contribute knowledge about the future spread of COVID-19 resulted in a flood of papers appearing on preprint servers [191], which will in due course be peer reviewed and some will be published. The pressure to speed up the peer-review process, in order to address the urgent challenge, may result in a compromise in the quality of both the review process and the science that is thereby published. In our screening process in Section 5, we scrutinised 29 peer-reviewed publications and 23 preprint articles. Of these, we found one published and potentially four preprint studies that offer credible insight into the climate-related SARS-CoV-2 and COVID-19 dynamics and epidemiology with a reasonable degree of confidence and rigour.

The general prevalence of climatologically-coupled seasonal signals and environmental variable modulation seen in the majority of other viral respiratory diseases creates the expectation for a similar effect on SARS-CoV-2 and in COVID-19 epidemiology. However, this virus and disease have only been spreading for 8 months. Observational evidence available to date has not yet been analysed sufficiently thoroughly to show that climate-related modulation is indeed a significant factor. The studies reviewed in Section 5 have aimed to find signs for such a signal, but a variety of methodological problems render a definitive conclusion premature.

The currently available time series do not capture a full annual cycle at any one location, or globally. The first studies appeared in late January on preprint servers (the majority of these are yet to be formally published as of mid-July 2020). As such, the initial reports looked for spatial variation in infectivity within a region and attempt to explain it in terms of associated variability in temperature, humidity or other environmental factors among these locations. Later studies could have benefitted from the larger datasets and a wider range of variation in the environmental drivers, resulting from the global spread, but became increasingly confounded by co-varying differences among the countries’ socio-economic conditions and pandemic responses. To date, the ‘global’ messages coming from the current body of COVID-19 research in general, and in respect to the environmental drivers of the disease in particular, do not equitably address the specific dynamics and considerations pertaining to the ‘Global South’. This is in part likely due to the slightly later arrival of the disease in the southern hemisphere. Thus, fewer southern hemisphere countries have suffered outbreaks of the same scale and severity (at the stage of assembling this manuscript) as the epidemics in the Far East, Europe and the United States. At the time of writing, the situation in some South American countries (such as Brazil and Peru) was deteriorating quickly. There is also a technical challenge in countries with relatively lower medical health research capacity, such as those in Africa [192]. The upshot is a circumstantially driven bias in the current literature which needs to be corrected, for several reasons. Neglecting the hemispheric disparities in knowledge regarding the role of environmental variables on SARS-CoV-2 and the modulation of the COVID-19 epidemic influences the discussion on the attribution of the reductions in cases. Northern countries are likely to move past peak daily infections coincidentally with the height of summer. It also neglects the urgent consideration of countries which are moving into winter. Importantly, many of the countries in the global south have already-stressed healthcare systems, and accurate modelling is critical in determining policy interventions for control measures to protect the lives of some of the world’s most vulnerable people. The collective global experience can provide a shortcut to knowledge and information regarding the role of environmental variables on SARS-CoV-2 biology and modulation of COVID-19 epidemiology and seasonality, applicable anywhere, by exploiting the latitudinal phasing of seasons to conduct research in all climates zones simultaneously. This leads us to call for global collaboration on this topic.

Much of the work we reviewed failed to carefully consider the implications of the choice of available metrics for viral transmission. We deem *R*_0_ to be best suited for the purpose of finding environmental sensitivity and seasonal climatic signals; some parametric estimates from regression models can also work, provided that care is taken to constrain the cases to those that result from local transmissions up to the time when NPIs come into play. *R*_0_ is closely aligned with the SIR-SEIR model family, and can be derived from the inversion of time series of case rate data using these models (see below).

Due to the effects of the incubation period, it may be important to use daily data (rather than data averaged over a several days) and a suitable lag period for both environmental and test-result data incorporated in the analysis. In the case of a highly infectious disease such as COVID-19, manifesting in a densely populated location, the effect of daily weather variations on transmission mechanisms is likely to be overwhelmed by the sheer magnitude of exposure. It may be that environmental modulation is still an important factor in these circumstances, but may reflect in indoor environments rather than outdoor ambient conditions [193]. Once the disease spread begins to approach an equilibrium (*R_t_*~1), the environmental effect may become more apparent.

To date, studies that attempted to discern the effects of climate by comparing infection rates across regions with different climates have been compromised by the heterogeneities that exist across locations and times in terms of control measures applied [194], and social, economic and cultural conditions that affect the practise of social distancing. Most studies have omitted variables such as poverty, population size and demographics (particularly age frequencies of the populace), the density of the population and how much high-resolution clustering is present (such as in the informal settlements in many countries of the South), the degree of urbanisation, access to healthcare, mobility and migration, various types of comorbidities (e.g., TB, HIV, malnourishment), the effect of the Bacillus Calmette-Guérin(BCG) vaccine [195], and a plethora of additional influences which are still not well understood with regards to how they influence the unfolding of COVID-19 across the globe. Simple graphing of case numbers across time in relation to some of the potentially influential drivers (as for example permitted by the Our World in Data Coronavirus Pandemic Data Explorer) will help reveal which of the additional variables to admit into the analysis.

An important obstacle to finding the seasonal signal in the global COVID-19 data is to find a way to deal with the hemispheric disparity (gradient away from the equator) in out-of-phase climatic signals. Comparing the evolution of COVID-19 for northern hemisphere countries moving from winter to summer to its evolution in southern hemisphere countries moving from summer to winter provides a valuable opportunity to discern the signature of seasonality. However, such a comparison will remain compromised by short time series and can only fully fulfil its potential once both hemispheres have experienced a full annual seasonal cycle.

We have concluded that due to high values of *R*_0_ exhibited by SARS-CoV-2, seasonal climate modulation should not be relied on to significantly dampen the infection rate even in the midst of the northern hemisphere approaching summer. Should the disease persist several years into the future, however, under the condition of an increasing fraction of the population of a given region having immunity, it is likely that the COVID-19 will exhibit an increasingly clear seasonal cycle as evident in similar endemic human coronaviruses. Such insights will only be apparent after the main pandemic surge in 2020.

We suggest some avenues for progress in addressing the environmental sensitivity of the disease. In addition to regression and correlative empirical approaches (Section 5.4), non-linear methods can also be applied. These may include the use of extended Kalman filters and the inversion of compartment models. Extended Kalman filters are commonly used in data assimilation to infer parameters from high-dimensional input data sets. Recently, Pei et al. [196] applied an ensemble-adjusted Kalman filter to infer the differential spatial distribution of COVID-19 infection rates from empirical data collected across different counties in the USA, followed by their application in a SEIR model. It may be feasible to apply this technique to estimate the relative roles of non-pharmaceutical control measures and seasonality in determining the infection rate. Inverse modelling, particularly using SEIR-type models, can infer infection rates from case and testing data, as demonstrated for the Hubai Province in China [46]. Making use of large ensembles that ingest data from many locations and systematically explore various combinations of the forcings can potentially explore the relative sensitivities of infection rates to NPI control measures and seasonality.

We recommend the use of regression-type statistical analyses than can be adapted to accommodate many simultaneous driving variables, including both environmental and non-environmental factors, thereby removing confounding influences. These models also readily accept non-Gaussian error terms and can account for autocorrelation in time series. Lags between exposure and when an individual is confirmed as infected can be accommodated by distributed lag non-linear models [197,198]. These techniques rely on Generalised Additive Models (GAMs) for the flexible estimation of smooth responses and parametric terms. The recognition that disease dynamics may differ between locations for a multitude of reasons requires that ‘location’ be specified as random effect (notable examples involving COVID-19 include Carlton et al. [153] and Wilson [174]). Such approaches can be accommodated by longitudinal models (called panel regressions by economists) (sensu Gardiner et al. [199]), which regress the dependant variable (plus covariates and constraints) as a function of time. Care should be given to estimations of uncertainties around model predictions —such estimates of uncertainties are permitted by Markov Chain Monte Carlo (MCMC) approaches [42]. Knowing the uncertainties is necessary in assessing projections from competing models in the public policy space. Finally, multivariate approaches, such as Redundancy Analysis (RDA) or Constrained Correspondence Analysis (CCA), will also accept a creative assignment of a host of response and influential variables simultaneously, and can be employed when research is faced with many potentially contributing factors, each of which might explain a portion of the overall variability.

We noted a lamentable deficiency in the application of reproducible research practices in many of the publications we reviewed. Clear, precise reporting of data sources and quality, data screening practices, listings of the ancillary data sources used, a detailed account of the data processing and statistical procedures and software used, and the exact reporting of all relevant diagnostic and supporting statistics, tables and figures is essential, particularly in this global emergency, where published data and information are used operationally, and where robust guidance is most likely to emerge from meta-analyses of many studies. Lives, livelihoods, economies, and the public trust in science depend on rigour and reproducibility. It is thus incumbent upon global research organisations and agencies such as the World Health Organisation (WHO) and the World Meteorological Organisation (WMO) to provide leadership and guidance and to define best-practice protocols for the analysis of data and production of information. To this end, the WHO has produced a document entitled “A Coordinated Global Research Roadmap: 2019 Novel Coronavirus” [200]. Its scope is broad, and thus does not specifically address some of the issues raised in our review. The authors are aware [201] that at the time of writing, the WMO has agreed to set up a Task Team which will focus on the environmental aspects of the COVID-19 pandemic.

## 7. Conclusions

Datasets capturing even the first full seasonal cycle of COVID-19 incidence in one locality, region or globally are not yet available and it is not possible at this stage to conclude that a definitive and unequivocal signal of environmental modulation is apparent from the reviewed literature. However, there is some evidence that environmental drivers played a role in transmission in some regions and at some (early) stages of the pandemic. Under other circumstances, longer and denser datasets would be a minimum requirement to support a thorough statistical treatment to explore evidence of environmental modulation of the COVID-19 pandemic and epidemiological dynamics. Pressure for rapid answers and information has prompted impulsive and dubious forays into signal-finding missions, such as those that dominate the current body of literature that had accumulated to date (15 July 2020). Analyses based on space-for-time substitutions have been inconclusive, primarily due to lack of care taken to account for the effects of strong confounding variables, such as socio-economic influences and effects of NPIs, which exist between jurisdictions. In terms of the outcomes of the published work, most studies are insensitive to the idiosyncratic conditions unique to many Southern Hemisphere countries, rendering it challenging to transfer findings from north to south. Rigorous hypotheses, interrogation of assumptions, and careful selection and development of analytical approaches and statistical models are required to examine environmental signals in complex COVID-19 incidence datasets, especially prior to longer and denser time series data being available. In the interim, there is merit in comparisons of signals among contrasting locations at different scales, and with due consideration paid to the implementation of NPIs and other sources of ‘noise’. This outcome does not discount the role of environmental drivers in modulating the incidence or seasonality of person-to-person transfection mechanisms, or of the morbidity, severity and mortality associated with COVID-19 infections. However, these may become unequivocally discernable only at later stages of the pandemic in 2020 or 2021, and globally coordinated efforts to test this robustly are essential. 

## Figures and Tables

**Table 1:** A summary of modelling approaches applied to COVID-19.

Theoretical Basis	Advantages	Disadvantages	Examples
(a) Simple extrapolation of recent trends—linear or exponential	Few assumptions, nearly theory-free, easily updated as new data come in	Sensitive to data quality; unrealistic for projections more than a few timesteps into the future	Systrom and Vladeck [43]
(b) Phenomenological or parameterised models—fit a curve of predetermined form to cumulative case data	Few assumptions, good for explaining large-scale, multi-month patterns like ‘flattening the curve’	Inflexible and unresponsive to changes in circumstances, such as social distancing policy	Della Morte et al. [44], Roosa et al. [45]
(c) Compartment models (e.g., SIR, SEIR)	Classical epidemiological approach, semi-mechanistic	Relatively many parameters that are highly uncertain initially; needs lots of good data	Anastassopoulou et al. [46]
(d) Machine learning	Few assumptions other than data homogeneity and stationarity	Requires very large case datasets to be effective; no explicit mechanism	Ardabili et al. [47], Pinter et al. [48]
(e) Agent-based models—every person in a population is modelled	Allows a rich set of interpersonal interactions despite simple rules	Data and computationally intensive	Cuevas [49]

**Table 2:** Viability of the human coronavirus, HCoV 229E, as a function of time, temperature and humidity [93].

Relative Humidity	20 °C				6 °C	
	15 min	24 h	72 h	6 days	15 min	24 h
30%	87%	65%	>50%	n.d.	91%	65%
50%	90.9%	75%	>50%	20%	96.5%	80%
80%	55%	3%	0%	n.d.	104.8%	86%

n.d. = not detectable.

## Supplementary Materials

The following are available online at https://www.mdpi.com/1660-4601/17/16/5634/s1, Figure S1: Discipline backgrounds of authors whose publications were included in this review. Table S1: Studies that have aimed to establish links between SARS-CoV-2 infections and environmental variables, notably temperature and humidity. All studies have in common a finding that the transmission of the virus is enhanced under colder, dryer conditions. Table S2: Studies that have aimed to establish links between SARS-CoV-2 infections and environmental variables, notably temperature and humidity. All studies have in common a finding that the transmission of the virus is enhanced under colder, dryer conditions.

## References

[1] Wang C., Horby P.W., Hayden F.G., Gao G.F. (2020). A novel coronavirus outbreak of global health concern. Lancet.

[2] Nicola M., Alsafi Z., Sohrabi C., Kerwan A., Al-Jabir A., Iosifidis C., Agha M., Agha R. (2020). The socio-economic implications of the coronavirus pandemic (COVID-19): A review. Int. J. Surg..

[3] Kickbusch I., Leung G.M., Bhutta Z.A., Matsoso M.P., Ihekweazu C., Abbasi K. (2020). Covid-19: How a virus is turning the world upside down. Br. Med. J..

[4] Mahler D.G., Lakner C., Aguilar R.A.C., Wu H. (2020). The Impact of COVID-19 (Coronavirus) on Global Poverty: Why Sub-Saharan Africa Might be the Region Hardest Hit.

[5] Tanne J.H., Hayasaki E., Zastrow M., Pulla P., Smith P., Rada A.G. (2020). Covid-19: How doctors and healthcare systems are tackling coronavirus worldwide. Br. Med. J..

[6] Coronavirus Government Response Tracker. https://www.bsg.ox.ac.uk/research/research-projects/coronavirus-government-response-tracker.

[7] Shaman J., Kohn M. (2009). Absolute humidity modulates influenza survival, transmission, and seasonality. Proc. Natl. Acad. Sci. USA.

[8] Shaman J., Pitzer V.E., Viboud C., Grenfell B.T., Lipsitch M. (2010). Absolute humidity and the seasonal onset of influenza in the continental United States. PLoS Boil..

[9] Xu S., Li Y. (2020). Beware of the second wave of COVID-19. Lancet.

[10] Nassar M.S., Bakhrebah M.A., Meo S.A., Alsuabeyl M.S., Zaher W.A. (2018). Global seasonal occurrence of middle east respiratory syndrome coronavirus (MERS-CoV) infection. Eur. Rev. Med Pharmacol. Sci..

[11] Carleton T., Meng K.C. Causal Empirical Estimates Suggest COVID-19 Transmission Rates Are Highly Seasonal. https://github.com/emlan-ucsb/COVID-seasonality.

[12] Sajadi M.M., Habibzadeh P., Vintzileos A., Shokouhi S., Miralles-Wilhelm F., Amoroso A. (2020). Temperature, humidity, and latitude analysis to estimate potential spread and seasonality of coronavirus disease 2019 (COVID-19). JAMA Netw. Open.

[13] Healy J. (2003). Analysing health care systems performance: The story behind the statistics. Aust. N. Z. J. Public Health.

[14] Hirve S., Newman L.P., Paget J., Azziz-Baumgartner E., Fitzner J., Bhat N., Vandemaele K., Zhang W. (2016). Influenza seasonality in the tropics and subtropics—When to vaccinate?. PLoS ONE.

[15] Fieno J., Lloyd-Sherlock P. (2002). Healthcare Reform and Poverty in Latin America.

[16] Naicker S., Plange-Rhule J., Tutt R.C., Eastwood J.B. (2009). Shortage of healthcare workers in developing countries--Africa. Ethn. Dis..

[17] Atun R., De Andrade L.O.M., Almeida G., Cotlear D., Dmytraczenko T., Frenz P., García P., Gómez-Dantés O., Knaul F.M., Muntaner C. (2015). Health-system reform and universal health coverage in Latin America. Lancet.

[18] Hampshire K., Porter G., Owusu S.A., Mariwah S., Abane A., Robson E., Munthali A., Delannoy A., Bango A., Gunguluza N. (2015). Informal m-health: How are young people using mobile phones to bridge healthcare gaps in Sub-Saharan Africa?. Soc. Sci. Med..

[19] Kapata N., Ihekweazu C., Ntoumi F., Raji T., Chanda-Kapata P., Mwaba P., Mukonka V., Bates M., Tembo J., Corman V.M. (2020). Is Africa prepared for tackling the COVID-19 (SARS-CoV-2) epidemic. Lessons from past outbreaks, ongoing pan-African public health efforts, and implications for the future. Int. J. Infect. Dis..

[20] Gilbert M., Pullano G., Pinotti F., Valdano E., Poletto C., Boëlle P.-Y., D’Ortenzio E., Yazdanpanah Y., Eholie S.P., Altmann M. (2020). Preparedness and vulnerability of African countries against importations of COVID-19: A modelling study. Lancet.

[21] Velavan T.P., Meyer C.G. (2020). The COVID-19 epidemic. Trop. Med. Int. Health.

[22] Ji Y., Ma Z., Peppelenbosch M.P., Pan Q. (2020). Potential association between COVID-19 mortality and health-care resource availability. Lancet Glob. Health.

[23] Talisuna A., Okiro E.A., Yahaya A.A., Stephen M., Bonkoungou B., Musa E.O., Minkoulou E.M., Okeibunor J., Impouma B., Djingarey H.M. (2020). Spatial and temporal distribution of infectious disease epidemics, disasters and other potential public health emergencies in the World Health Organisation Africa region, 2016–2018. Glob. Health.

[24] Rodriguez-Morales A.J., Gallego V., Escalera-Antezana J.P., Mendez C.A., Zambrano L.I., Franco-Paredes C., Suárez J.A., Rodriguez-Enciso H.D., Balbin-Ramon G.J., Savio-Larriera E. (2020). COVID-19 in Latin America: The implications of the first confirmed case in Brazil. Travel Med. Infect. Dis..

[25] Steingart K.R., Sohn H., Schiller I.A., Kloda L., Boehme C.C., Pai M., Dendukuri N. (2013). Xpert^®^ MTB/RIF assay for pulmonary tuberculosis and rifampicin resistance in adults. Cochrane Database Syst. Rev..

[26] Gelaw Y.A., Williams G.M., Magalhães R.J.S., Gilks C.F., Assefa Y. (2019). HIV prevalence among tuberculosis patients in sub-Saharan Africa: A systematic review and meta-analysis. AIDS Behav..

[27] Grant A., Charalambous S., Fielding K., Day J.H., Corbett E.L., Chaisson R.E., De Cock K.M., Hayes R., Churchyard G. (2005). Effect of routine isoniazid preventive therapy on tuberculosis incidence among HIV-Infected men in South Africa. J. Am. Med. Assoc..

[28] Corbett E.L., Marston B., Churchyard G.J., De Cock K.M. (2006). Tuberculosis in sub-Saharan Africa: Opportunities, challenges, and change in the era of antiretroviral treatment. Lancet.

[29] Adenis A., Valdes A., Cropet C., McCotter O.Z., Derado G., Couppié P., Chiller T., Nacher M. (2018). Burden of HIV-associated histoplasmosis compared with tuberculosis in Latin America: A modelling study. Lancet Infect. Dis..

[30] Blanco J.L., Ambrosioni J., Garcia F., Martínez E., Soriano A., Mallolas J., Miró J.M. (2020). COVID-19 in HIV Investigators COVID-19 in patients with HIV: Clinical case series. Lancet HIV.

[31] Jiang H., Zhou Y., Tang W. (2020). Maintaining HIV care during the COVID-19 pandemic. Lancet HIV.

[32] Pang Y., Liu Y., Du J., Gao J., Li L. (2020). Impact of COVID-19 on tuberculosis control in China. Int. J. Tuberc. Lung Dis..

[33] Preiser W., Van Zyl G., Dramowski A. (2020). COVID-19: Getting ahead of the epidemic curve by early implementation of social distancing. S. Afr. Med. J..

[34] Liu Y., Yan L.-M., Wan L., Xiang T.-X., Le A., Liu J.-M., Peiris M., Poon L.L., Zhang W. (2020). Viral dynamics in mild and severe cases of COVID-19. Lancet Infect. Dis..

[35] Patel R., Babady N.E., Theel E.S., Storch G.A., Pinsky B.A., George K.S., Smith T.C., Bertuzzi S. (2020). Report from the American Society for Microbiology COVID-19 International Summit, 23 March 2020: Value of Diagnostic Testing for SARS–CoV-2/COVID-19. mBio.

[36] Ye F., Xu S., Rong Z., Xu R., Liu X., Deng P., Liu H., Xu X. (2020). Delivery of infection from asymptomatic carriers of COVID-19 in a familial cluster. Int. J. Infect. Dis..

[37] Grifoni A., Weiskopf D., Ramirez S.I., Mateus J., Dan J.M., Moderbacher C.R., Rawlings S.A., Sutherland A., Premkumar L., Jadi R.S. (2020). Targets of T cell responses to SARS-CoV-2 coronavirus in humans with COVID-19 disease and unexposed individuals. Cell.

[38] Leon D.A., Shkolnikov V.M., Smeeth L., Magnus P., Pechholdová M., Jarvis C.I. (2020). COVID-19: A need for real-time monitoring of weekly excess deaths. Lancet.

[39] Yao Y., Pan J., Liu Z., Meng X., Wang W., Kan H., Wang W. (2020). No association of COVID-19 transmission with temperature or UV radiation in Chinese cities. Eur. Respir. J..

[40] Şahin M. (2020). Impact of weather on COVID-19 pandemic in Turkey. Sci. Total Environ..

[41] Xie J., Zhu Y. (2020). Association between ambient temperature and COVID-19 infection in 122 cities from China. Sci. Total Environ..

[42] Heesterbeek J.A.P., Anderson R., Andreasen V., Bansal S., De Angelis D., Dye C., Eames K.T.D., Edmunds W.J., Frost S.D., Funk S. (2015). Modeling infectious disease dynamics in the complex landscape of global health. Science.

[43] Systrom K., Vladeck T. Rt COVID-19. https://rt.live/.

[44] Della Morte M., Orlando D., Sannino F. (2020). Renormalization group approach to pandemics: The COVID-19 case. Front. Phys..

[45] Roosa K., Lee Y., Luo R., Kirpich A., Rothenberg R., Hyman J., Yan P., Chowell G. (2020). Real-time forecasts of the COVID-19 epidemic in China from February 5th to February 24th, 2020. Infect. Dis. Model..

[46] Anastassopoulou C., Russo L., Tsakris A., Siettos C. (2020). Data-based analysis, modelling and forecasting of the COVID-19 outbreak. PLoS ONE.

[47] Ardabili S.F., Mosavi A., Ghamisi P., Ferdinand F., Varkonyi-Koczy A.R., Reuter U., Rabczuk T., Atkinson P.M. (2020). COVID-19 outbreak prediction with machine learning. medRxiv.

[48] Pinter G., Felde I., Mosavi A., Ghamisi P., Gloaguen R. (2020). COVID-19 pandemic prediction for Hungary; a hybrid machine learning approach. Mathematics.

[49] Cuevas E. (2020). An agent-based model to evaluate the COVID-19 transmission risks in facilities. Comput. Boil. Med..

[50] Shi P., Dong Y., Yan H., Li X., Zhao C., Liu W., He M., Tang S., Xi S. (2020). The impact of temperature and absolute humidity on the coronavirus disease 2019 (COVID-19) outbreak-evidence from China. medRxiv.

[51] Neher R.A., Dyrdak R., Druelle V., Hodcroft E.B., Albert J. (2020). Potential impact of seasonal forcing on a SARS-CoV-2 pandemic. Swiss Med. Wkly..

[52] Flaxman S., Mishra S., Gandy A., Unwin H., Coupland H., Mellan T., Zhu H., Berah T., Eaton J., Perez Guzman P. (2020). Estimating the Number of Infections and the Impact of Nonpharmaceutical Interventions on COVID-19 in 11 European Countries.

[53] Lofgren E.T., Fefferman N.H., Naumov Y.N., Górski J., Naumova E.N. (2006). Influenza seasonality: Underlying causes and modeling theories. J. Virol..

[54] Du Prel J.-B., Puppe W., Gröndahl B., Knuf M., Weigl J.A.I., Schaaff F., Schmitt H.-J. (2009). Are meteorological parameters associated with acute respiratory tract Infections?. Clin. Infect. Dis..

[55] Stewart P.D.S. (2016). Seasonality and selective trends in viral acute respiratory tract infections. Med. Hypotheses.

[56] Drexler J.F., Corman V.M., Drosten C. (2014). Ecology, evolution and classification of bat coronaviruses in the aftermath of SARS. Antivir. Res..

[57] Killerby M.E., Biggs H.M., Haynes A., Dahl R.M., Mustaquim D., Gerber S.I., Watson J.T. (2018). Human coronavirus circulation in the United States 2014–2017. J. Clin. Virol..

[58] Price R.H.M., Graham C., Ramalingam S. (2019). Association between viral seasonality and meteorological factors. Sci. Rep..

[59] Cohen J. (2020). Sick time. Science.

[60] Moriyama M., Hugentobler W.J., Iwasaki A. (2020). Seasonality of respiratory viral infections. Annu. Rev. Virol..

[61] Martinez M.E. (2018). The calendar of epidemics: Seasonal cycles of infectious diseases. PLoS Pathog..

[62] Fisman D.N. (2012). Seasonality of viral infections: Mechanisms and unknowns. Clin. Microbiol. Infect..

[63] Viboud C., Alonso W.J., Simonsen L. (2006). Influenza in tropical regions. PLoS Med..

[64] Saunders-Hastings P., Krewski D. (2016). Reviewing the history of pandemic influenza: Understanding patterns of emergence and transmission. Pathogens.

[65] Al-Tawfiq J.A., Memish Z.A. (2016). Drivers of MERS-CoV transmission: What do we know?. Expert Rev. Respir. Med..

[66] Skowronski D.M., Astell C., Brunham R.C., Low D.E., Petric M., Roper R.L., Talbot P.J., Tam T., Babiuk L. (2005). Severe Acute Respiratory Syndrome (SARS): A Year in Review. Annu. Rev. Med..

[67] Dowell S.F., Ho M.S. (2004). Seasonality of infectious diseases and severe acute respiratory syndrome–what we don’t know can hurt us. Lancet Infect. Dis..

[68] Kissler S., Tedijanto C., Goldstein E., Grad Y.H., Lipsitch M. (2020). Projecting the transmission dynamics of SARS-CoV-2 through the postpandemic period. Science.

[69] Moore K.A., Lipsitch M., Barry J.M., Osterholm M.T. (2020). Part 1: The Future of the COVID-19 Pandemic: Lessons Learned from Pandemic Influenza. COVID-19.

[70] Chan K.H., Peiris J.S.M., Lam S.Y., Poon L.L., Yuen K.-Y., Seto W.H. (2011). The Effects of temperature and Relative humidity on the viability of the SARS coronavirus. Adv. Virol..

[71] Tellier R. (2009). Aerosol transmission of influenza a virus: A review of new studies. J. R. Soc. Interface.

[72] Lowen A.C., Mubareka S., Steel J., Palese P. (2007). Influenza virus transmission is dependent on relative humidity and temperature. PLOS Pathog..

[73] Lowen A., Palese P. (2009). Transmission of influenza virus in temperate zones is predominantly by aerosol, in the tropics by contact: A hypothesis. PLoS Curr..

[74] Tamerius J.D., Shaman J., Alonso W.J., Bloom-Feshbach K., Uejio C.K., Comrie A., Viboud C. (2013). Environmental predictors of seasonal influenza epidemics across temperate and tropical climates. PLoS Pathog..

[75] Baker R.E., Mahmud A.S., Metcalf C.J.E. (2018). Dynamic response of airborne infections to climate change: Predictions for varicella. Clim. Chang..

[76] Bourouiba L. (2016). A Sneeze. N. Engl. J. Med..

[77] Yang W., Elankumaran S., Marr L.C. (2012). Relationship between humidity and influenza a viability in droplets and implications for influenza’s seasonality. PLOS ONE.

[78] Cole E.C., Cook C.E. (1998). Characterization of infectious aerosols in health care facilities: An aid to effective engineering controls and preventive strategies. Am. J. Infect. Control..

[79] Atkinson J., Chartier Y., Pessoa-Silva C.L., Jensen P., Li Y., Seto W.H., WHO (2009). WHO Guidelines Approved by the Guidelines Review Committee. Natural Ventilation for Infection Control in Health-Care Settings.

[80] Liu L., Wei J., Li Y., Ooi A. (2016). Evaporation and dispersion of respiratory droplets from coughing. Indoor Air.

[81] Tellier R., Li Y., Cowling B.J., Tang J.W. (2019). Recognition of aerosol transmission of infectious agents: A commentary. BMC Infect. Dis..

[82] Lowen A.C., Steel J. (2014). Roles of humidity and temperature in shaping influenza seasonality. J. Virol..

[83] Zhang Y., Bambrick H., Mengersen K., Tong S., Hu W. (2018). Using google trends and ambient temperature to predict seasonal influenza outbreaks. Environ. Int..

[84] Nielsen J., Mazick A., Glismann S., Mølbak K. (2011). Excess mortality related to seasonal influenza and extreme temperatures in Denmark, 1994-2010. BMC Infect. Dis..

[85] Jaakkola K., Saukkoriipi A., Jokelainen J., Juvonen R., Kauppila J., Vainio O., Ziegler T., Rönkkö E., Jaakkola J.J.K. (2014). Decline in temperature and humidity increases the occurrence of influenza in cold climate. Environ. Health.

[86] Rabenau H., Cinatl J., Morgenstern B., Bauer G., Preiser W., Doerr H.W. (2004). Stability and inactivation of SARS coronavirus. Med. Microbiol. Immunol..

[87] Sauerbrei A., Wutzler P. (2008). Testing thermal resistance of viruses. Arch. Virol..

[88] Bean B., Moore B.M., Sterner B., Peterson L.R., Gerding D.N., Balfour H.H. (1982). Survival of influenza viruses on environmental surfaces. J. Infect. Dis..

[89] Boone S.A., Gerba C.P. (2007). Significance of fomites in the spread of respiratory and enteric viral disease. Appl. Environ. Microbiol..

[90] Casanova L.M., Jeon S., Rutala W.A., Weber D.J., Sobsey M.D. (2010). Effects of air temperature and relative humidity on coronavirus survival on surfaces. Appl. Environ. Microbiol..

[91] Kampf G., Todt D., Pfaender S., Steinmann E. (2020). Persistence of coronaviruses on inanimate surfaces and their inactivation with biocidal agents. J. Hosp. Infect..

[92] McDevitt J., Rudnick S., First M., Spengler J. (2010). Role of absolute humidity in the inactivation of influenza viruses on stainless steel surfaces at elevated temperatures. Appl. Environ. Microbiol..

[93] Geller C., Varbanov M., Duval R.E. (2012). Human coronaviruses: Insights into environmental resistance and its influence on the development of new antiseptic strategies. Viruses.

[94] Sizun J., Yu M., Talbot P. (2000). Survival of human coronaviruses 229E and OC43 in suspension and after drying onsurfaces: A possible source of hospital-acquired infections. J. Hosp. Infect..

[95] Otter J., Donskey C., Yezli S., Douthwaite S., Goldenberg S.D., Weber D. (2016). Transmission of SARS and MERS coronaviruses and influenza virus in healthcare settings: The possible role of dry surface contamination. J. Hosp. Infect..

[96] Kramer A., Schwebke I., Kampf G. (2006). How long do nosocomial pathogens persist on inanimate surfaces? A systematic review. BMC Infect. Dis..

[97] Lowen A.C., Steel J., Mubareka S., Palese P. (2008). High temperature (30 °C) blocks aerosol but not contact transmission of influenza virus. J. Virol..

[98] Ellwanger J.H., Chies J.A.B. (2018). Wind: A neglected factor in the spread of infectious diseases. Lancet Planet. Health.

[99] Peci A., Winter A.-L., Li Y., Gnaneshan S., Liu J., Mubareka S., Gubbay J.B. (2019). Effects of absolute humidity, relative humidity, temperature, and wind speed on influenza activity in Toronto, Ontario, Canada. Appl. Environ. Microbiol..

[100] Firestone S.M., Cogger N., Ward M.P., Toribio J.-A.L.M.L., Moloney B., Dhand N.K. (2012). The influence of meteorology on the spread of influenza: Survival analysis of an equine influenza (A/H3N8) Outbreak. PLoS ONE.

[101] Yusuf S., Piedimonte G., Auais A., Demmler G., Krishnan S., Van Caeseele P., Singleton R., Broor S., Parveen S., Avendaño L. (2007). The relationship of meteorological conditions to the epidemic activity of respiratory syncytial virus. Epidemiol. Infect..

[102] Hervás D., Reina J., Hervás J.A. (2012). Meteorologic conditions and respiratory syncytial virus activity. Pediatr. Infect. Dis. J..

[103] Guo Q., Dong Z., Zeng W., Ma W., Zhao D., Sun X., Gong S., Xiao J., Li T., Hu W. (2018). The effects of meteorological factors on influenza among children in Guangzhou, China. Influenza Other Respir. Viruses.

[104] Adler R.F., Gu G., Sapiano M., Wang J.J., Huffman G.J. (2017). Global precipitation: Means, variations and trends during the satellite era (1979–2014). Surv. Geophys..

[105] Pica N., Bouvier N.M. (2012). Environmental factors affecting the transmission of respiratory viruses. Curr. Opin. Virol..

[106] Murray E.L., Klein M., Brondi L., McGowan J.E., Van Mels C., Brooks W.A., Kleinbaum D., Goswami D., Ryan P.B., Bridges C.B. (2011). Rainfall, household crowding, and acute respiratory infections in the tropics. Epidemiol. Infect..

[107] Paynter S., Weinstein P., Ware R.S., Lucero M.G., Tallo V., Nohynek H., Barnett A., Skelly C., Simoes E.A.F., Sly P.D. (2012). Sunshine, rainfall, humidity and child pneumonia in the tropics: Time-series analyses. Epidemiol. Infect..

[108] Chew F.T., Doraisingham S., Ling A.E., Kumarasinghe G., Lee B.W. (1998). Seasonal trends of viral respiratory tract infections in the tropics. Epidemiol. Infect..

[109] Idani E., Dastoorpoor M., Goudarzi G., Khanjani N. (2016). Severe outbreaks of respiratory syndromes following autumn rainfall in Khuzestan, Iran. Arch. Iran. Med..

[110] Duan S.-M., Zhao X.-S., Wen R.-F., Huang J.-J., Pi G.-H., Zhang S.-X., Han J., Bi S.-L., Ruan L., Dong X.-P. (2003). Stability of SARS coronavirus in human specimens and environment and its sensitivity to heating and UV irradiation. Biomed. Environ. Sci..

[111] Darnell M.E., Subbarao K., Feinstone S.M., Taylor D.R. (2004). Inactivation of the coronavirus that induces severe acute respiratory syndrome, SARS-CoV. J. Virol. Methods.

[112] Bedell K., Buchaklian A.H., Perlman S. (2016). Efficacy of an automated multiple emitter whole-room ultraviolet-C disinfection system against coronaviruses MHV and MERS-CoV. Infect. Control. Hosp. Epidemiol..

[113] Sagripanti J.-L., Lytle C.D. (2007). Inactivation of influenza virus by solar radiation. Photochem. Photobiol..

[114] Weber T.P., Stilianakis N.I. (2008). Inactivation of influenza a viruses in the environment and modes of transmission: A critical review. J. Infect..

[115] Eccles R. (2002). An Explanation for the seasonality of acute upper respiratory tract viral infections. Acta Oto-Laryngol..

[116] Kudo E., Song E., Yockey L.J., Rakib T., Wong P.W., Homer R.J., Iwasaki A. (2019). Low ambient humidity impairs barrier function and innate resistance against influenza infection. Proc. Natl. Acad. Sci. USA.

[117] Cannell J., Vieth R., Umhau J.C., Holick M.F., Grant W.B., Madronich S., Garland C.F., Giovannucci E. (2006). Epidemic influenza and vitamin D. Epidemiol. Infect..

[118] Liu P.T., Stenger S., Li H., Wenzel L., Tan B.H., Krutzik S.R., Ochoa M.T., Schauber J., Wu K., Meinken C. (2006). Toll-Like receptor triggering of a vitamin D-mediated human antimicrobial response. Science.

[119] Moan J.E., Dahlback A., Ma L., Juzeniene A. (2009). Influenza, solar radiation and vitamin D. Dermat-Endocrinology.

[120] Yamshchikov A.V., Desai N.S., Blumberg H.M., Ziegler T.R., Tangpricha V. (2009). Vitamin D for treatment and prevention of infectious diseases: A systematic review of randomized controlled Trials. Endocr. Pract..

[121] Shaman J., Jeon C.Y., Giovannucci E., Lipsitch M. (2011). Shortcomings of vitamin D-based model simulations of seasonal influenza. PLoS ONE.

[122] Ciencewicki J., Jaspers I. (2007). Air pollution and respiratory viral infection, inhalation. Toxicology.

[123] Schwartz J. (1994). Air pollution and daily mortality: A review and meta analysis. Environ. Res..

[124] Clay K., Lewis J., Severnini E. (2018). Pollution, infectious disease, and mortality: Evidence from the 1918 Spanish influenza pandemic. J. Econ. Hist..

[125] Clifford H.D., Perks K.L., Zosky G.R. (2015). Geogenic PM10 exposure exacerbates responses to influenza infection. Sci. Total Environ..

[126] Ye Q., Fu J.-F., Mao J.H., Shang S.-Q. (2016). Haze is a risk factor contributing to the rapid spread of respiratory syncytial virus in children. Environ. Sci. Pollut. Res..

[127] Peng R.D., Dominici F., Pastor-Barriuso R., Zeger S.L., Samet J.M. (2005). Seasonal analyses of air pollution and mortality in 100 US Cities. Am. J. Epidemiol..

[128] Cichowicz R., Wielgosiński G., Fetter W. (2017). Dispersion of atmospheric air pollution in summer and winter season. Environ. Monit. Assess..

[129] Cheong K., Ngiam J., Morgan G.G., Pek P.P., Tan B.Y.-Q., Lai J.W., Koh J.M., Ong M., Ho A.F.W. (2019). Acute health impacts of the Southeast Asian transboundary haze problem—A review. Int. J. Environ. Res. Public Health.

[130] Auler A.C., Cássaro F.A.M., da Silva V.O., Pires L.F. (2020). Evidence that high temperatures and intermediate relative humidity might favor the spread of COVID-19 in tropical climate: A case study for the most affected Brazilian cities. Sci. Total Environ..

[131] Da Silva F.L., Gomes M.D.A., Da Silva A.P.L., De Sousa S.C., De Souza M.F.S., Da Silva G.L.P. (2020). Correlation between meteorological factors and COVID-19 infection in the Belem Metropolitan Region. medRxiv.

[132] Figueiredo A.M., Daponte-Codina A., Figueiredo D.C.M.M., Vianna R.P.T., de Lima K.C., Gil-García y.E. (2020). Factors associated with the incidence and mortality from COVID-19 in the autonomous communities of Spain. Gac Sanit.

[133] Neto R.A.A., Melo G.C. (2020). Correlation between weather, population size and COVID-19 pandemic: A study of Brazilian capitals. J. Health Boil. Sci..

[134] Prata D.N., Rodrigues W., Bermejo P.H. (2020). Temperature significantly changes COVID-19 transmission in (sub) tropical cities of Brazil. Sci. Total Environ..

[135] Rodrigues S.A., Dal Pai A., Grotto R.M.T., Sarnighausen V.C.R. (2020). Meteorological variables associations and the occurrence of COVID-19 in the City of São Paulo, Brazil. Revista Ibero-Americana de Ciências Ambientias.

[136] Velásquez R.M.A., Lara J.V.M. (2020). Gaussian approach for probability and correlation between the number of COVID-19 cases and the air pollution in Lima. Urban Clim..

[137] Ward M., Xiao S., Zhang Z. (2020). The role of climate during the COVID-19 epidemic in New South Wales, Australia. Transbound. Emerg. Dis..

[138] Bashir M.F., Ma B., Bilal K.B., Bashir M.A., Tan D., Bashir M. (2020). Correlation between climate indicators and COVID-19 pandemic in New York, USA. Sci. Total Environ..

[139] Tosepu R., Gunawan J., Effendy D.S., Ahmad L.O.A.I., Lestari H., Bahar H., Asfian P. (2020). Correlation between weather and Covid-19 pandemic in Jakarta, Indonesia. Sci. Total Environ..

[140] Gupta S., Raghuwanshi G.S., Chanda A. (2020). Effect of weather on COVID-19 spread in the US: A prediction model for India in 2020. Sci. Total Environ..

[141] Liu J., Zhou J., Yao J., Zhang X., Li L., Xu X., He X., Wang B., Fu S., Niu T. (2020). Impact of meteorological factors on the COVID-19 transmission: A multi-city study in China. Sci. Total Environ..

[142] Poirier C., Luo W., Majumder M.S., Liu D., Mandl K., Mooring T., Santillana M. (2020). The role of environmental factors on transmission rates of the COVID-19 Outbreak: An initial assessment in two spatial scales. SSRN Electron. J..

[143] Dee D.P., Uppala S.M., Simmons A.J., Berrisford P., Poli P., Kobayashi S., Andrae U., Balmaseda M.A., Balsamo G., Bauer D.P. (2011). The ERA-interim reanalysis: Configuration and performance of the data assimilation system. Q. J. R. Meteorol. Soc..

[144] Jahangiri M., Jahangiri M., Najafgholipour M. (2020). The sensitivity and specificity analyses of ambient temperature and population size on the transmission rate of the novel coronavirus (COVID-19) in different provinces in Iran. Sci. Total Environ..

[145] Gao M., Zhou Q., Zhang S., Yung K.K.L., Guo Y. (2020). Non-linear modulation of COVID-19 transmission by climate conditions. SSRN Electron. J..

[146] Guo X.-J., Zhang H., Zeng Y.-P. (2020). Transmissibility of COVID-19 and its association with temperature and humidity. Eur. PMC.

[147] Gupta A., Gharehgozli A. (2020). Developing a machine learning framework to determine the spread of COVID-19. SSRN Electron. J..

[148] Jamil T., Alam I.S., Gojobori T., Duarte C.M. (2020). No evidence for temperature-dependence of the COVID-19 epidemic. medRxiv.

[149] Jia J., Ding J., Liu S., Liao G., Li J., Duan B., Wang G., Zhang R. (2020). No evidence for temperature-dependence of the covid-19 epidemic. medRxiv.

[150] Jebril N. (2020). Predict the transmission of COVID-19 under the effect of air temperature and relative humidity over the year in Baghdad, Iraq. SSRN Electron. J..

[151] Luo W., Majumder M.S., Liu D., Poirier C., Mandl K.D., Lipsitch M., Santillana M. (2020). The role of absolute humidity on transmission rates of the COVID-19 outbreak. medRxiv.

[152] Wang M., Jiang A., Gong L., Luo L., Guo W., Li C., Zheng J., Li C., Yang B., Zeng J. (2020). Temperature significant change COVID-19 Transmission in 429 cities. medRxiv.

[153] Carleton T., Cornetet J., Huybers P., Meng K., Proctor J. (2020). Ultraviolet radiation decreases COVID-19 growth rates: Global causal estimates and seasonal implications. SSRN Electron. J..

[154] Caspi G., Shalit U., Kristensen S.L., Aronson D., Caspi L., Rossenberg O., Shina A., Caspi O. (2020). Climate effect on COVID-19 spread rate: An online surveillance tool. medRxiv.

[155] Ficetola G.F., Rubolini D. (2020). Climate affects global patterns of COVID-19 early outbreak dynamics. medRxiv.

[156] Merow C., Urban M.C. (2020). Seasonality and uncertainty in COVID-19 growth rates. medRxiv.

[157] Notari A. (2020). Temperature dependence of COVID-19 transmission. arxiv.

[158] Oliveiros B., Caramelo L., Ferreira N.C., Caramelo F. (2020). Role of temperature and humidity in the modulation of the doubling time of COVID-19 cases. medRxiv.

[159] Sahafizadeh E., Sartoli S. (2020). High temperature has no impact on the reproduction number and new cases of COVID-19 in Bushehr, Iran. medRxiv.

[160] Skutsch M., Dobler C., McCall M.B.B., Ghilardi A., Salinas M., McCall M.K., Sanchez F. (2020). The association of UV with rates of COVID-19 transmission and deaths in Mexico: The possible mediating role of vitamin D. medRxiv.

[161] Kotsiou O.S., Kotsios V.S., Lampropoulos I., Zidros T., Zarogiannis S.G., Gourgoulianis K.I. (2020). High temperature slows coronavirus disease 2019 transmission rate: A within and among country analysis. Res. Sq..

[162] Leung N.Y., Bulterys M.A., Bulterys P.L. (2020). Predictors of COVID-19 incidence, mortality, and epidemic growth rate at the country level. medRxiv.

[163] Lolli S., Chen Y.-C., Wang S.-H., Vivone G. (2020). Impact of meteorology and air pollution on Covid-19 pandemic transmission in Lombardy region, Northern Italy. Res. Sq..

[164] Chen B., Liang H., Yuan X., Hu Y., Xu M., Zhao Y., Zhang B., Tian F., Zhu X. (2020). Roles of meteorological conditions in COVID-19 transmission on a worldwide scale. medRxiv.

[165] Lin J., Huang W., Wen M., Li D., Ma S., Hua J., Hu H., Yin S., Qian Y., Chen P. (2020). Containing the spread of coronavirus disease 2019 (COVID-19): Meteorological factors and control strategies. Sci. Total Environ..

[166] Rahman A., Hossain G., Singha A.C., Islam S., Islam A. (2020). A retrospective analysis of influence of environmental/air temperature and relative humidity on SARS-CoV-2 outbreak. PrePrints.

[167] Wu Y., Jing W., Liu J., Ma Q., Yuan J., Wang Y., Du M., Liu M. (2020). Effects of temperature and humidity on the daily new cases and new deaths of COVID-19 in 166 countries. Sci. Total Environ..

[168] Bannister-Tyrrell M., Meyer A., Faverjon C., Cameron A. (2020). Preliminary evidence that higher temperatures are associated with lower incidence of COVID-19, for cases reported globally up to 29th February 2020. medRxiv.

[169] Tobías A., Molina T. (2020). Is temperature reducing the transmission of COVID-19?. Environ. Res..

[170] Wang J., Tang K., Feng K., Lv W. (2020). High Temperature and high humidity reduce the transmission of COVID-19. SSRN Electron. J..

[171] Xu H., Yan C., Fu Q., Xiao K., Yu Y., Han D., Wang W., Cheng J. (2020). Possible environmental effects on the spread of COVID-19 in China. Sci. Total Environ..

[172] Qi H., Xiao S., Shi R., Ward M.P., Chen Y., Tu W., Su Q., Wang W., Wang X., Zhang Z. (2020). COVID-19 transmission in Mainland China is associated with temperature and humidity: A time-series analysis. Sci. Total Environ..

[173] Ujiie M., Tsuzuki S., Ohmagari N. (2020). Effect of temperature on the infectivity of COVID-19. Int. J. Infect. Dis..

[174] Wilson D.J. (2020). Weather, Social Distancing, and the Spread of COVID-19.

[175] Ahmadi M., Sharifi A., Dorosti S., Ghoushchi S.J., Ghanbari N. (2020). Investigation of effective climatology parameters on COVID-19 outbreak in Iran. Sci. Total Environ..

[176] Alipio M. (2020). Do latitude and ozone concentration predict COVID-2019 cases in 34 countries?. SSRN Electron. J..

[177] Bhattacharjee S. (2020). Statistical investigation of relationship between spread of coronavirus disease (COVID-19) and environmental factors based on study of four mostly affected places of China and five mostly affected places of Italy. arxiv.

[178] Briz-Redón Á., Serrano-Aroca Á. (2020). A spatio-temporal analysis for exploring the effect of temperature on COVID-19 early evolution in Spain. Sci. Total Environ..

[179] Sobral M.F.F., Duarte G.B., da Penha Sobral A.I.G., Marinho M.L.M., de Souza Melo A. (2020). Association between climate variables and global transmission of SARS-CoV-2. Sci. Total Environ..

[180] Mollalo A., Vahedi B., Rivera K.M. (2020). GIS-based spatial modelling of COVID-19 incidence rate in the continental United States. Sci. Total Environ..

[181] Pirouz B., Haghshenas S.S., Pirouz B., Haghshenas S.S., Piro P. (2020). Development of an assessment method for investigating the impact of climate and urban parameters in confirmed cases of COVID-19: A new challenge in sustainable development. Int. J. Environ. Res. Public Health.

[182] Arias-Reyes C., Zubieta-DeUrioste N., Poma-Machicao L., Aliaga-Raudan F., Carvajal-Rodriguez F., Dutschmann M., Schneider-Gasser E.M., Zubieta-Calleja G., Soliz J. (2020). Does the pathogenesis of SARS-CoV-2 virus decrease at high-altitude?. Respir. Physiol. Neurobiol..

[183] Bukhari Q., Jameel Y. (2020). Will coronavirus pandemic diminish by summer?. SSRN Electron. J..

[184] Gunthe S.S., Swain B., Patra S.S., Amte A. (2020). On the global trends and spread of the COVID-19 outbreak: Preliminary assessment of the potential relation between location-specific temperature and UV index. J. Public Health.

[185] Iqbal N., Fareed Z., Shahzad F., He X., Shahzad U., Lina M. (2020). Nexus between COVID-19, temperature and exchange rate in Wuhan city: New findings from partial and multiple wavelet coherence. Sci. Total Environ..

[186] Araujo M.B., Naimi B. (2020). Spread of SARS-CoV-2 Coronavirus likely to be constrained by climate. medRxiv.

[187] Coro G. (2020). A global-scale ecological niche model to predict SARS-CoV-2 coronavirus infection rate. Ecol. Model..

[188] Carlson C.J., Chipperfield J., Benito B.M., Telford R.J., O’Hara R.B. (2020). Species distribution models are inappropriate for COVID-19. Nat. Ecol. Evol..

[189] Baker R.E., Yang W., Vecchi G.A., Metcalf C.J.E., Grenfell B.T. (2020). Susceptible supply limits the role of climate in the early SARS-CoV-2 pandemic. Science.

[190] Wan X., Cheng C., Zhang Z. (2020). Early transmission of COVID-19 has an optimal temperature but late transmission decreases in warm climate. medRxiv.

[191] Kwon D. (2020). How swamped preprint servers are blocking bad coronavirus research. Nature.

[192] Beran D., Byass P., Gbakima A., Kahn K., Sankoh O., Tollman S., Witham M., Davies J.I. (2017). Research capacity building—Obligations for global health partners. Lancet Glob. Health.

[193] Morawska L., Cao J. (2020). Airborne transmission of SARS-CoV-2: The world should face the reality. Environ. Int..

[194] COVID-19 Health System Response Monitor. https://www.covid19healthsystem.org.

[195] Escobar L.E., Molina-Cruz A., Barillas-Mury C. (2020). BCG vaccine protection from severe coronavirus disease 2019 (COVID-19). Proc. Natl. Acad. Sci. USA.

[196] Pei S., Kandula S., Shaman J. (2020). Differential Effects of Intervention Timing on COVID-19 Spread in the United States. medRxiv.

[197] Gasparrini A., Guo Y., Hashizume M., Lavigne E., Zanobetti A., Schwartz J., Tobías A., Tong S., Rocklöv J., Forsberg B. (2015). Mortality risk attributable to high and low ambient temperature: A multicountry observational study. Lancet.

[198] Liu C., Chen R., Sera F., Vicedo-Cabrera A.M., Guo Y., Tong S., Coelho M.S., Saldiva P.H., Lavigne E., Matus P. (2019). Ambient particulate air pollution and daily mortality in 652 cities. N. Engl. J. Med..

[199] Gardiner J.C., Luo Z., Roman L.A. (2009). Fixed effects, random effects and GEE: What are the differences?. Stat. Med..

[200] WHO—A Coordinated Global Research Roadmap: 2019 Novel Coronavirus. https://www.who.int/blueprint/priority-diseases/key-action/Coronavirus_Roadmap_V9.pdf.

[201] Shumake-Guillemot J. (2020). Personal communication.

