# Supplementary material for: Winter Is Coming: A Southern Hemisphere Perspective of the Environmental Drivers of SARS-CoV-2 and the Potential Seasonality of COVID-19
Source: Int J Environ Res Public Health. 2020 Aug 5;17(16):5634. doi: 10.3390/ijerph17165634 (PMC7459895; doi:10.3390/ijerph17165634)
Supplement: Supplementary file 1 [file ijerph-17-05634-s001.zip › IJERPH Supplement Tables S1 and S2 - revision FINAL.docx]

**Table S1.** Publications that have aimed to establish links between SARS-CoV-2 infections and environmental variables, notably temperature and humidity.

*NPI: non-pharmaceutical interventions. † Studies based on parameter estimates related to some aspect of the initial portion of exponential curves (or other parametric models) that model daily new infections as a function of time. § Study applies* R_0_*, SEIR model estimates (see next Section 5.3). ‡ Studies offering moderate to strong support for the hypothesis that environmental variables modulate the rate of transmission of COVID-19 (see Section 5.5 in main paper).*

|  | **Countries** | **Time span** | **Lags** | **Independent variable(s)** | **Dependent variable(s)** | **Statistical approach** | **Additional influences** | **NPI** | **Reference** |
| --- | --- | --- | --- | --- | --- | --- | --- | --- | --- |
|  | Iran | 19 February to 22 March 2020 | None | Infection days to end of the study period, average temperature, mean precipitation, humidity, wind speed, mean solar radiation | Daily COVID-19 cases | Correlation, sensitivity analysis between variables evaluated based on the Partial correlation coefficient (PCC) and Sobol'-Janson methods, visual mapping | Population density, intra-provincial movement, classification of provinces based on the De Martonne method | No | Ahmadi et al. (2020) |
|  | Tibet, Bolivia, Ecuador | 19 March to 7 April 2020 | None | Altitude | Daily COVID-19 cases | Visual mapping | None | No | Arias-Reyes et al. (2020) |
|  | Five Brazilian cities | 13 March to 13 April 2020 | None | Mean, maximum and minimum daily temperatures, absolute humidity, rainfall | Daily new and cumulative COVID-19 cases, contamination rate (per 100,000 habitants) | Principal component analyses (PCA), canonical correlation, linear regression | None | No | Auler et al. (2020) |
|  | Global | Not specified |  | Assumes same humidity sensitivity as influenza, HCoV-OC43 and HCoV-HKU1 | *R_0_* from the SARS-CoV-2 pandemic (using USA case data) | SIRS model simulations, parameterized with SARS-CoV-2 *R_0_* vs humidity effects from other respiratory viral diseases | None | No | Baker et al. (2020) |
|  | New York City, USA | 1 March to 12 April 2020 | Lags of 3 and 4 days | Mean, maximum, minimum temperature, rainfall, humidity, wind speed, air quality | Daily new and total COVID-19 cases | Kendall and Spearman correlation | None | No | Bashir et al. (2020) |
|  | Provinces of Spain | 25 February to 28 March 2020 | 5 days | Mean, maximum minimum temperature | Daily cumulative COVID-19 cases | Mixed models (3rd order polynomial) | Population density, age, number of travellers, number of companies | No | Briz-Redón and Serrano-Aroca (2020) |
|  | 72 Global locations | To end of March 2020 | None | Temperature, precipitation, and elevation | Average “rates of infected people” (not well defined) | Maximum entropy-based  ecological niche model | CO_2_ and population density | No | Coro (2020) |
|  | 10 Chinese Provinces, Australia, Belgium, Egypt, Finland, Iran, Italy, Philippines, South Korea | 31 December 2019 to 29 February 2020 | None | Maximum, minimum temperature | Unspecified, but probably number of cases | Student’s t-test or Mann-Whitney U test of pairs of countries | Ongoing human-to-human transmission | No | Del Rio and Camacho-Ortiz (2020) |
|  | 21 Countries and French administrative regions | 15 February to 3 week in March 2020 | None | Temperature | Daily COVID-19 cases | ARIMA model | None | No | Demongeot et al. (2020) |
|  | Brazil | 15 March to 22 April 2020 | None | Temperature | Daily COVID-19 cases | Spearman correlation, multiple regression | Population density, number people >65 years old, structure of health services | No | Figueiredo et al. (2020) |
|  | Global (85 locations, listed in Gunthe et al., 2020, Table 1) | 2 February to 10 March 2020 | None | Mean, maximum, minimum, relative humidity, UV index, precipitation, cloud cover | Number of cases | Scatterplots, “various statistical fits” | None | No | Gunthe et al. (2020) |
|  | 50 States of the USA | 1 January to 9 April 2020 | None | Temperature, relative humidity, absolute humidity derived via Clausius Clapeyron equation | Daily COVID-19 cases | Manual classification akin to a correlation by ranks | None | No | Gupta et al. (2020b) |
|  | India | To 27 April 2020 | None | Temperature, rainfall, actual evapotranspiration, solar radiation, specific humidity, wind speed | Total COVID-19 cases | Pearson correlation, linear regression, log-linear GAM | Altitude, population density | No | Gupta et al. (2020a) |
|  | Wuhan, China | 21 January to 31 March 2020 | None | Temperature | Daily COVID-19 cases | Wavelet Transform Coherence, Partial Wavelet Coherence, Multiple Wavelet Coherence | None | No | Iqbal et al. (2020a) |
|  | Global | To 5 June 2020 | None | Averages of maximum and minimum temperature, daylight hours | Total COVID-19 cases | Linear regression | Population density | No | Iqbal et al. (2020b) |
|  | 31 Iranian provinces | 15 February to the 22 March 2020 | None | Temperature | Daily COVID-19 cases | Receiver operating characteristics (ROC) curves | Population density | No | Jahangiri et al. (2020) |
|  | Global, excl. excluding China, South Korea, Iran and Italy | To 28 March 2020 | None | Temperature, humidity | ‘Ratios of rate ratios’ of  cumulative counts | Weighted random-effects regression (uni- and multivariate) | Latitude, school closures, restrictions of mass gatherings, and measures of social distancing | No | Jüni et al. (2020) |
|  | 30 Chinese cities | 5 January to 22 March 2020 | 3 days | Temperature, diurnal temperature range, relative humidity, absolute humidity via Clausius Clapeyron equation | Daily COVID-19 cases | Polynomial regression, GLMs with random effects (mixed) models | Migration scale index | Yes | Liu et al. (2020) |
|  | Global | 25 March to 19 April 2020 | None | Temperature | COVID-19 case data at 6 day intervals, total cases, active cases | Spearman and Kendall correlations, log-linear regressions | Population density | No | Mandal and Panwar (2020) |
|  | Counties of continental USA | 22 January to 9 April 2020 | None | Mean, maximum, minimum temperature, precipitation | COVID-19 incidence rate  (unspecified) | Various regression models | 35 environmental, socioeconomic, topographic, and demographic variables | Yes | Mollalo et al. (2020) |
|  | Temperate Northern Hemisphere regions | Not specified |  | Climate sensitivities taken from HKU1, NL63, OC43 and 229E | None | Seasonality estimated from other CoV and applied to SEIR mod. of SARS-CoV-2 | Migration rates | No | Neher et al. (2020) |
|  | Brazil | 18 April to 1 May 2020 | None | Temperature, dew point, average humidity, wind speed | Total COVID-19 cases | Spearman correlation | Population density | No | Neto and de Melo (2020) |
|  | Spain, provincial level | 13 March to 11 April 2020 | 3 lags | Temperature, humidity, hours of sunshine | Daily COVID-19 cases | ‘Seemingly Unrelated Regression’ | Population density, GDP per capita, percentage of older adults in population, mass transit systems | No | Paez et al. (2020) |
|  | Five provinces of Italy | 29 February to 29 March 2020 | None | Temperature, relative humidity | Daily COVID-19 cases smoothed by 5 and 8 day moving averages | Pearson correlation | Climatic regions, cases adjusted for number of tests made | No | Passerini et al. (2020) |
|  | Italy | 14 February to 14 March 2020 | None | Temperature, relative humidity, wind speed | Daily COVID-19 cases | Multivariate linear regression | Urban variables such as population density | Yes | Pirouz et al. (2020) |
|  | Chinese provinces, incl. 345 cities; also Iran, Italy, Japan, Singapore, South Korea | 22 January to 26 February 2020 | None | Temperature, absolute humidity | Proxy R_t_ from daily COVID-19 cases | Loess regression | Mobility | Yes | Poirier et al. (2020) |
|  | 27 Brazilian capital cities | 27 February to 1 April 2020 | None | Temperature | Daily COVID-19 cases | GAM, polynomial regression | Population density and size | No | Prata et al. (2020) |
|  | 30 Chinese provinces | 1 December 2019 to 20 January 2020 | 14 days | Temperature, relative humidity | Daily COVID-19 cases | GAM |  | No | Qi et al. (2020) |
|  | 9 Cities in Turkey | February to 10 April 2020 | None | Temperature, humidity, dew point, wind speed. | Daily COVID-19 cases (not explicitly stated) | Spearman correlation | Population density | No | Şahin (2020) |
|  | All affected countries | January to February 2020 | None | Temperature, humidity | Presence of significant community transmission | Maps of temperature and infected countries | None | No | Sajadi et al. (2020) |
|  | China, Italy, USA | From December 2019 | None | Temperature, relative humidity, wind speed, atmospheric pressure, air pollution | Total COVID-19 cases | Pearson correlation | Median population age, population density | No | Scafetta (2020) |
|  | All affected countries | 1 December 2019 to 30 March 2020 | None | Mean, maximum, minimum temperature, precipitation | Daily COVID-19 cases | Panel regression | Population density, time of exposure | No | Sobral et al. (2020) |
|  | Kuala Lumpur, Malaysia | 11 March to 21 April 2020 | None | Relative humidity, temperature, wind speed, solar radiation; air pollutants data incl. PM10, PM2.5, SO_2_, NO_2_, O_3_, CO | Daily COVID-19 cases | Paired-samples t-test, Wilcoxon test, Spearman correlation, multiple linear regression | None | No | Suhaimi et al. (2020) |
|  | Barcelona region, Spain | 25 February to 5 April 2020 | 6 days | Maximum temperature | Daily COVID-19 cases | GLM (with autocorrelation) | Weekends, the lockdown period | No | Tobias and Molina (2020) |
|  | Jakarta, Indonesia | January to 29 March 2020 | None | Mean, maximum, minimum temperature, humidity, rainfall | Daily COVID-19 cases | Spearman correlation | None | No | Tosepu et al. (2020) |
|  | Japan’s 47 prefectures | 5 January to 16 March 2020 | None | Temperature | Cumulative number of cases per million | GLM | Population density, mobility, age | No | Ujie et al. (2020) |
|  | Lima, Peru | From March to 9 April 2020 | None | CO, NO_2_, O_3_, SO_2_, PM10, PM2.5 | ‘Infections’ (not specified) | ‘Gaussian Process Regression’ and cross-validation using a neural network | None | No | Velásquez and Lara (2020) |
|  | New South Wales, Australia | January to end of March 2020 | Exponential moving averages of 10 to 21 days | Rainfall, temperature, relative humidity | Daily COVID-19 cases | Correlation, multivariate GAM | None | No | Ward et al. (2020) |
|  | 166 Countries | 27 March to 22 April 2020 | 14 days | Temperature, relative humidity, dew point, wind speed | Daily COVID-19 cases | GAM | Age, Global Health Security Index, Human Development Index, population density | No | Wu et al. (2020) |
|  | 33 Chinese locations | 29 January to 15 February 2020 | Lag from 0-7 days | Temperature, relative humidity, atmospheric pressure, wind speed, SO_2_, NO_2_, PM10 and PM2.5, carbon monoxide, ozone | Daily COVID-19 cases | GLM | None | No | Xu et al. (2020a) |
| §  ‡ | China | Early January to early March 2020 | None | Temperature, relative humidity, UV radiation averaged for time period | *R_0_* | Multiple regression | None | No | Yao et al. (2020) |
|  | 122 Cities in mainland China | 23 January to 29 February 2020 | 14 days | Temperature, relative humidity, air pressure, wind speed | Daily COVID-19 cases | GAM | None | Yes | Zhu and Xie (2020) |

**Table S2.** Preprint studies that have aimed to establish links between SARS-CoV-2 infections and environmental variables, notably temperature and humidity.

*NPI: non-pharmaceutical interventions. † Studies based on parameter estimates related to some aspect of the initial portion of exponential curves (or other parametric models) that model daily new infections as a function of time. § Study applies* R_0_*, SEIR model estimates (see next Section 5.3). ‡ Studies offering moderate to strong support for the hypothesis that environmental variables modulate the rate of transmission of COVID-19 (see Section 5.5 in main paper).*

|  | **Countries** | **Time span** | **Lags** | **Independent variable(s)** | **Dependent variable(s)** | **Statistical approach** | **Additional influences** | **NPI** | **Reference** |
| --- | --- | --- | --- | --- | --- | --- | --- | --- | --- |
|  | Philippines | 16 March to 2May 2020 | None | Maximum temperature | Daily recovery rate | Pearson and Spearman correlation | None | No | Acosta et al. (2020) |
|  | Australia, Brazil, Canada, Germany, Italy, New Zealand, Singapore, South Africa, Sweden, UK | 22 January to 30 April 2020 | Lag of 14 days | Minimum, maximum temperature, humidity, wind speed | Cumulative COVID-19 cases | Spearman correlation | Hospital beds/1000, Hospital occupancy, lockdown stringency index, USD per capita index, population density | Yes | Adeyemi et al. (2020) |
|  | Three cities in each of Spain, Italy, USA | 26 February to 4 March 2020 | None | Temperature, relative humidity, wind velocity | Cumulative COVID-19 cases | Regression | Population density, travel restrictions. | Yes | Adhikari et al. (2020) |
|  | 70 Cities, regions globally | 18 January to 24 April 2020 | None | Average monthly humidity, minimum and maximum temperature | Cumulative COVID-19 cases | GLM | Population density | No | Ahmed et al. (2020) |
|  | 34 countries (see Alipio, 2020, Table 4) | Not specified | None | Latitude, ozone | COVID-19 cases (unspecified) | Kendall correlation, multiple regression | None | No | Alipio (2020) |
|  | All countries with five or more cases | Up to 10 March 2020 | None | Mean, maximum, minimum temperature, precipitation | COVID-19 cases | Machine learning, ecological niche models | None | No | Araújo and Naimi (2020) |
|  | Belgium,. Brazil, Canada, China, France, Germany, India, Iran, Italy, Peru, Russia, Spain, Turkey, UK | February to 13 May 2020 | Modelled lag (not unambiguously specified) | Average temperature, relative humidity | Daily COVID-19 cases | GAM | None | Yes | Awasthi et al. (2020) |
|  | 162 countries and several regions in China | Up to 20 March 2020 | None | Average temperature (4 week period). Solar irradiance (solar elevation angle) | Daily COVID-19 cases, case rate change (during 11 days after reaching 100 cases) | Correlation | None | No | Bäcker (2020) |
|  | Global to date of study | Up to 29 February 2020 | None | Temperature | Cumulative number of COVID-19 | GLM | Age, capacity to detect emerging diseases | No | Bannister-Tyrrell et al. (2020.) |
|  | 186 countries | Up to 13 April 2020 | None | Annual average temperature, annual relative humidity | Cumulative COVID-19 cases | Correlation | Population density, infant mortality rate, gender ratio, population age structure, life expectancy, human development index, socioeconomic classification, BCG vaccination, lockdown (none, localized or national lockdown), number of days between the first case and lockdown and the number of cases at lockdown | No | Bellali et al. (2020) |
|  | Global (information unavailable) | 1 January to 31 March 2020 | None | 3 month average temperature, precipitation, latitude | Daily COVID-19 cases | Correlation | Population, population density, median age, GCG vaccine policy, | No | Bezabih et al. (2020) |
|  | “four mostly affected places of China and five mostly affected places of Italy” | Up to 13 March 2020 | None | Maximum temperature, relative humidity, wind speed | Daily new COVID-19 cases | Pearson correlation | None | No | Bhattacharjee (2020) |
|  | Wuhan | Not clearly specified | None | Temperature, humidity, precipitation | COVID-19 cases | Not specified | None | No | Bu et al. (2020) |
|  | Global to date of study | Cases reported up to 19 March 2020 | None | Temperature, relative and absolute humidity, wind speed | Daily COVID-19 cases | Visual display of total case counts relative to humidity and temperature | None | No | Bukhari and Jameel (2020) |
|  | China | 25 January to 29 February 2020 | Lag of 6 days | PM2.5, PM10, SO_2_, NO_2_ CO, O_3._ daily mean ambient temperature, relative humidity and wind velocity | Daily and cumulative COVID-19 cases | GAMMS | None | No | Cao et al. (2020) |
|  | Global | The period 22 January and15 March 2020 | None | Temperature, specific humidity | COVID-19 cases | Panel (longitudinal) regression | Population density, various socio-economic and country-specific variables | No | Carlton and Meng (2020) |
| †  ‡ | 173 countries (3235 geospatial units) | 10 January to 10 April 2020 | Lag of 3 days | UV, temperature, humidity, precipitation | Daily cumulative case number growth rate | Distributed lag panel regression models | Population density, socio-economic variation among locations | Yes | Carlton et al. (2020) |
| † | Global, excluding China | Up to 19 March 2020 | None | Temperature, humidity, dew point, precipitation, wind speed | Replication rate, slope the logarithmic curve of confirmed cases, rate of spread, doubling time | Pearson and Spearman correlation | Population data | No | Caspi et al. (2020) |
|  | 430 Cities, districts across China | 20 January to 11 March 2020 | Four time points delays | Temperature, relative humidity, dew point, wind speed | Daily COVID-19 cases | GLM, polynomial regressions | None | No | Chen et al. (2020a) |
|  | China | From January to February | None | Daily average, maximum temperature, minimum temperature, relative humidity, precipitation, air pressure, SO_2_, CO, O_3_, NO_2_, PM2.5, PM10 | Daily COVID-19 cases | GLM | None | No | Chen et al. (2020b) |
| † | Chile (121 cities) organised by climate zone | 23 February to 26 April 2020 | None | Weekly average temperature, relative humidity, atmospheric pressure, windspeed | Weekly case rate | Correlation and GLM | Population size | No | Correa-Araneda et al. (2020) |
|  | Italy (21 regions) and USA (3142 counties) | Up to 7 April 2020 | Lags of 7-14 days | Averaged specific humidity, temperature, UV, precipitation | Cumulative case numbers (8 days) | GLM and GAM | Population size, embarkations (air travel) | Yes | Corripio and Raso (2020) |
|  | Brazil (Belém Metropolitan Region) | 18^th^ March to the 6^th^ of May 2020 | None | Average minimum, maximum temperature, relative humidity, precipitation, wind  speed | Daily COVID-19 cases | Correlation | Gender and gender ratio, populations size, population density, age distribution, | Yes | da Silva et al. (2020) |
|  | China | 20 January to 5 May 2020 | Lag of 14 days | Minimum, maximum and average temperature, humidity, air pressure, PM10, PM2.5, NO_2_, CO_2_ | Daily COVID-19 cases | Correlation, Wavelet analysis | None | No | Damette and Goutte (2020) |
|  | India | 18 March to 30 April 2020 | None | 14 day moving average n  temperature, relative humidity, solar radiation, rainfall, wind speed, PM2.5, PM10, SO_2_, NO_2_  and CO | Daily COVID-19 cases | Spearman correlation, GAM | None | Yes | Das and Das Chatterjee (2020) |
|  | 204 Countries globally | From January to April 2020 | None | Average minimum, maximum and average temperatures for January to April 2020 | Cumulative total cases critical cases, recoveries to April 2020 | Correlation, GLM | None | No | Das et al. (2020) |
|  | Angola, Burundi, Ethiopia, Malawi, Mauritania, Mozambique, Nicaragua, Papua New Guinea, South Sudan, Uganda, Yemen, Zimbabwe, Nepal, Syria | December 2019 to 26 April 2020 | None | Temperature (classified as ‘hot’ and ‘cold’) | Total cases as on 26 April 2020 | None | Population density | No | de Gennaro (2020) |
|  | India, 5 cities | To 20 May 2020 | 3 days | Daily temperature and relative humidity | Daily COVID-19 cases | Linear mixed model (controlling for city) | None | No | Dixit et al. (2020) |
| †  ‡ | 79 Countries, regions (not named) | 22 January to 31 March 2020 | None | Temperature, specific humidity, PM2.5 | Mean daily growth rate from exponential phase of the growth curve | Linear mixed models | Population size and density, per capita health expenditure, age structure, air pollution | Yes | Ficetola and Rubolini (2020) |
|  | Spain, Madrid and Catalonia regions | April 2020 | None | Mean April temperature | COVID-19 cases (unspecified) | Pearson correlation | Population density | No | Franco and Galo-Fernandez (2020a) |
|  | Spain, Catalonian “comarcas” | April 2020 | None | Mean April temperature | COVID-19 cases (unspecified) | Spearman correlation | Population density | No | Franco and Galo-Fernandez (2020b) |
| § | ‘35 selected countries across the globe’ | From 26 April 2020 | None | 7 Day mean temperature and relative humidity | *R_0_* | Linear regression (not clearly specified) | Age structure and patterns of contact in each country (unspecified) | Yes | Gao et al. (2020) |
| § | Wuhan and 10 major Chinese cities outside Hubei Province | 18 January to 13 February 2020 | None | Temperature, humidity | *R_0_* | Linear regression | None | Yes | Guo et al. (2020) |
| §  ‡ | USA | 14 January to 16 April 2020 | None | Mean temperature and humidity (over unspecified period); PM2.5 | *R_0_* | Multiple regression, support vector machines, decision trees | Median age, population density, per capita GDP | Yes | Gupta and Gharehgozli (2020) |
|  | India, 9 cities | 30 January to 4 June 2020 | 7, 10, 12, 14, 16 days | Maximum, minimum, mean temperature, diurnal temperature range, dew point, mean relative humidity, range in relative humidity, wind speed | Daily COVID-19 cases | Spearman correlation | Population density, elevation | No | Gupta and Pradhan (2020) |
|  | Bangladesh, 6 districts | 8 March to 17 May 2020 | None | Daily temperature, humidity, wind speed | Daily COVID-19 cases | Maps, Spearman and Kendal correlation | None | No | Hasan and Siddik (2020) |
|  | Japan, 19 prefectures | 15 to 25 March 2020 | None | Temperature, absolute humidity | ‘Spread duration’ | Regression | Population density | No | Hirata et al. (2020) |
|  | China, 250 cities | December 2019 to 15 February 2020 | None | Daily temperature, relative humidity, precipitation, wind speed, air pressure, and visibility | ‘number of confirmed COVID-19 infected cases’ (not specified) | Pearson correlation | Population density, mobility | No | Huang et al. (2020) |
|  | Global, 116 countries | 8 January to 12 March 2020 | 7 and 14 day lags | Temperature, humidity, wind speed, UV-index | ‘number of COVID-19 cases’ (not specified) | Multilevel mixed effects negative .binomial regression models | None | No | Islam et al. (2020) |
| §  † | All affected countries, and provinces of China | 31 December 2019 to 26 March 2020 | None | Temperature | Exponential rate parameters from infection case vs time curves, *R_0_* | Regression | None | Yes | Jamil et al. (2020) |
|  | Iraq | 25 February to 17 April 2020 | Lag 3 day | Temperature, relative humidity | *R_t_* | Some kind of visual eyeballing | None | No | Jebril (2020) |
| §  † | Chinese Provinces, excl. Qinghai, Tibet, Hong Kong, Macao, Taiwan | Up to 19 February 2020 | None | A ‘comprehensive meteorological index’ incl. ‘air index’, temperature, precipitation, relative humidity, wind power | *R_0_*, *β* (the contact rate) | Correlation (unspecified) | Migration | Yes | Jia (2020) |
|  | Global, 46 worst affected countries | 31 December 2019 to 29 March 2020 | None | Daily temperature | Daily COVID-19 cases, rates as time from first case to 200 cases | Pearson and Spearman correlation, Student’s *t*-test | None | No | Kotsiou et al. (2020) |
| ‡ | Global, 205 countries and territories | To 2 May 2020 | 1 Week and 1 month prior (mean of influential variable) | Mean, maximum, minimum temperature, atmospheric pressure, mean relative humidity, total rainfall and/or snowmelt, mean visibility, mean wind speed, total days with snow, total days with thunderstorm, total days with fog | Total number of COVID-19 cases, cumulative number of cases at 28 days after the first reported case | Uni- and multivariate negative binomial regression | Age structure, sex ratio, population size, population density, urban population, GDP per capita, Human Development Index, number of airports, number of air travellers per annum; body mass index, obesity, diabetes, smoking, hospital beds, physicians, health expenditure, PM2.5, O_3_, household air pollution, Climate Risk Index (2018), number of COVID-19 tests performed | Yes | Leung et al. (2020) |
| ‡ | China, Hong Kong, Singapore, | 17 to 25 March 2020 | 12 ± 3 days | Daily temperature and humidity | Transmission rates obtained from SEIR models | Multiple regression | Country-level effects | Yes | Lin et al. (2020) |
|  | Lombardy, Italy | 8 March to 19 June 2020 | 20 days | Minimum, mean, maximum of temperature, dew point, relative humidity, absolute humidity, wind speed, pressure; NO_2_, PM2.5 | Daily ICU case anomaly | Spearman and Kendal correlation | None (ICU case anomaly data avoids many issues associated with testing and reporting) | No | Lolli et al. (2020) |
|  | Each province in China (incl. Hong Kong), and Japan, Singapore, South Korea, Taiwan, Thailand | 23 January to 10 February 2020 | None | Temperature, absolute humidity | Proxy *R_t_* | Linear regression | None | No | Luo et al. (2020) |
|  | India’s Maharashtra, Rajasthan and Kashmir regions | 9 March to 27 May 2020 | None | Temperature | Daily COVID-19 cases | Correlation, regression | None | No | Meraj et al. (2020) |
| †  ‡ | 128 Countries and 98 states or provinces (not named) | Up to 13 April 2020 | None | Temperature, relative humidity, UV radiation | Exponential growth rate of early COVID-19 cases | Bayesian methods, including random (per country) effects | Indoor aggregation, proportion elderly | Yes | Merow and Urban (2020) |
|  | 15 German state capitals | 1 March to 8 May 2020 | None | Mean temperature, humidity | Daily COVID-19 cases | None, visual examination of graphs | Population density | Yes | Munnangi et al. (2020) |
|  | Spanish autonomous communities | October 2019 to 15 April 2020 | None | Temperature, relative humidity, UV-radiation | Cumulative COVID-19 cases during the previous 14 days, total cases, newly diagnosed cases, hospital admissions, intensive care unit admissions | Spearman correlation, linear regression | None | No | Muñoz-Cacho et al. (2020) |
|  | USA counties | 22 January to 13 June 2020 | None | Temperature | Daily COVID-19 cases | Linear regression, generalized method of moments | Population density | No | NoghaniBehambari et al. (2020) |
| † | Global | 23 January to 1 March 2020 | None | Mean temperature for March | Parameters estimated from curves fitted to the infection vs. time | Exponential model, GLM, other parametric models | None | No | Notari (2020) |
| † | Mainland China | 23 January to 1 March 2020 | None | Temperature, humidity, precipitation, wind speed | Doubling time | Exponential and linear models | Cultural aspects, policies adopted to contain the virus | No | Oliveiros et al. (2020) |
|  | Global, ~230 cities | January to April 2020 | None | Mean diurnal temperature range, temperature seasonality; humidity | ‘COVID-19 cases’ (not specified) | Boosted regression trees | Annual Parasite Index, international travel | No | Pramanik et al. (2020) |
|  | Australia, China, France, Germany, Iran, Italy, Japan, Singapore, South Korea, Spain, Switzerland, USA | 31 January to 28 February 2020 | None | Temperature, relative humidity | Daily confirmed COVID-19 cases | Linear regression | None | No | Rahman et al. (2020a) |
|  | Global, 149 countries | 1 January to 10 May 2020 | None | Maximum, minimum, mean temperatures, and temperature extreme | Rate of spread of COVID-19 (total confirmed cases/total test performed) × 100) | Partial correlation analysis and linear mixed effect model | Population density, population growth rate, GDP growth rate, GDP per capita, life expectancy, % population over 60 years, health expenditure, physicians per thousand people; number of threatened species, forested area, CO_2_ emission, area % of the forested area protected | No | Rahman et al. (2020b) |
|  | Global | January to April 2020 | None | Temperature, specific humidity, dew point temperature, air pressure, wind speed and total precipitation | Log monthly COVID-19 cases | Pearson correlation | None | No | Rasul and Balzter (2020) |
|  | Global, 118 countries (popl. > 5 mil.) | 2 March to 30 June 2020 | None | Monthly temperature | Monthly COVID-19 cases | Correlation, non-linear curve fitting | Population density | No | Ren and Chen (2020) |
|  | Italy | 24 February to 15 April 2020 | None | Mean February temperature, relative humidity, air pollution | Daily COVID-19 cases | Bayesian model averaging | Population density, policy lags, number of tests, health spending, proportions of young and old in population, social mobility | No | Rios and Gianmoena (2020) |
|  | São Paulo, Brazil | 26 February to 6 April 2020 | None | Temperature, relative humidity, atmospheric pressure, wind speed | Daily COVID-19 cases | Spearman correlation, GLM with negative binomial distribution | None | No | Rodrigues et al. (2020) |
|  | Global | 31 December 2019 to 3 April 2020 | None | Temperature climatologies | COVID-19 cases (unspecified) | t-test of categories of temperatures globally, mapping | Population size | Yes | Roy (2020) |
| † | Bushehr, Iran | From April to 12 June 2020 | None | Monthly mean temperature | Daily COVID-19 cases, *R_t_*, parametric estimates | None | None | No | Sahafizadeh and Sartoli (2020) |
|  | Global | 23 January to 11 April 2020 | 5-days | Temperature | Daily COVID-19 cases | Wilcoxon test | None | No | Sethwala et al. (2020) |
|  | Spain, incl. Castilla y Leon, Castilla-La Mancha, Catalonia, Madrid | February to 17 April 2020 | None | Daily mean temperature, PM2.5 | Daily COVID-19 cases | Pearson, Spearman and Kendall correlations; panel and quantile regressions | None | No | Shahzad et al. (2020) |
|  | 31 Provinces in mainland China, incl. Wuhan city | 20 January to 25 February 2020 | None | Temperature, absolute humidity | Daily confirmed COVID-19 cases | Regression | None | No | Shi et al. (2020) |
|  | India, incl. Maharashtra, Delhi, Rajasthan, Gujarat, Tamilnadu, and Madhya Pradesh | From April 2020 | 7-days | Mean temperature, maximum relative humidity, maximum wind speed | Daily COVID-19 cases | Kendall and Spearman correlation | None | Yes | Singh and Agarwal (2020) |
| † | Mexico, 45 cities | 26 February to 3 May 2020 | None | Daily maximum temperature and humidity, UV-radiation | Weekly increment in COVID‐19 cases, transmission rates (slope of log-case vs. time per city) | Pearson correlation, multiple regression | Altitude | No | Skutsch et al. (2020) |
|  | 57 Countries | 9 January to 25 March 2020 | None | Daily mean temperature and relative humidity | Daily COVID-19 cases | Quadratic and linear regressions | None | No | Sobur et al. (2020) |
|  | Japanese prefectures | From January 2020 | None | Monthly mean temperature, wind speed, air pressure, relative humidity, sunshine; maximum UV index; total sunshine duration and precipitation | Cumulative COVID-19 cases | Random-effects meta-regression | Population density | No | Takagi et al. (2020) |
|  | Global, 138 countries | 22 January to 17 May 2020 | None | Duration of sunshine, minimum and maximum temperature | Daily COVID-19 cases | Spearman correlation, linear regression | Population density, median age, Global Health Security | No | Thangariyal et al. (2020) |
| †  ‡ | Global | 1 January to 11 March 2020 | None | Daily and monthly mean temperature and precipitation | Cumulative COVID-19 cases, transmission rates estimated by logistic regression for each locality | GAM | Population density, GDP, policy intervention | Yes | Wan et al. (2020) |
|  | 100 Chinese cities | To 23 January 2020 | None | Temperature, relative humidity | *R_t_* | Panel regressions (mixed model) | GDP per capita, population density, number of hospital beds, fraction of population over 65 | No | Wang et al. (2020a) |
|  | China and “26 overseas countries” | 20 January to 4 February 2020 | None | Mean, maximum, minimum temperature | Cumulative number of COVID-19 cases | GLM | None | No | Wang et al. (2020b) |
|  | Global, 116 countries/territories | December 2019 to May 2020 | None | Maximum temperature, relative humidity, rainfall, sunshine, UV-radiation | Monthly COVID-19 cases | Correlation, non-linear regression | Latitude | No | Wen and Chen (2020) |
|  | China | January to February 2020 | None | Mean temperature, specific humidity, UV-radiation | Daily COVID-19 cases | None | None | No | Wen et al. (2020) |
| †  ‡ | USA, county-level | Late January to 28 June 2020 |  | Daily maximum temperature, minimum temperature, precipitation, snowfall | Rate of daily new and cumulative COVID-19 cases | Panel local projections estimator | Google Mobility Reports; Dallas Fed Mobility and Engagement Index; nursing home population size; potential contemporaneous reverse causality; growth in testing | Yes | Wilson (2020) |
|  | Global | 12 December 2019 to 22 April 2020 | None | Daily minimum and maximum temperature, humidity, precipitation, snowfall, moon illumination, sunlight hours, UV index, cloud cover, wind speed and direction, air pressure; O_3_, NO_2_, SO_2_, PM2.5 | *R_t_* | Linear models | Population density; social distancing interventions; various location-specific fixed effects | Yes (?) | Xu et al. (2020b) |
|  | China, 120 cities | 15 January to 18 March 2020 | 0, 3, 7 and 14 day lags | Mean temperature and  diurnal range, relative humidity, wind velocity, air pressure, precipitation, hours of sunshine; PM2.5, NO_2_, SO_2_, CO, O_3_ | Daily COVID-19 cases | Spearman correlation, uni- and multivariate negative binomial GLM | None | No | Zhou et al. (2020) |
|  | China, 27 provincial capitals, 4 metropolitan cities | 1 February to 31 March 2020 | None | Temperature, humidity, visibility, wind speed, air pressure; aerosol data | Daily COVID-19 cases | Compound natural factor models | Vegetation data | No | Zuo et al. (2020) |

References

1. Acosta DS, Alquizar ML, Alexes Junio CJ, Van Buladuco M (2020). Correlational analysis of hot weather and number of recovery of the pandemic Coronavirus in The Philippines. *SSRN,* DOI: 10.2139/ssrn.3597254.
2. Adeyemi S, Yakutcan U, Demir E (2020). A statistical assessment of association between meteorological parameters and COVID-19 pandemic in 10 countries: the devil is in the detail. *Research Gate (Preprint),* available at: https://www.researchgate.net/profile/Usame_Yakutcan/publication/342313160_A_statistical_assessment_of_association_between_meteorological_parameters_and_COVID-19_pandemic_in_10_countries_the_devil_is_in_the_detail/links/5eecbd0a458515814a6ad77b/A-statistical-assessment-of-association-between-meteorological-parameters-and-COVID-19-pandemic-in-10-countries-the-devil-is-in-the-detail.pdf
3. Adhikari A, Ghosh S, Sen MM, Adhikari R (2020). Models of transmission of COVID-19 with time under the influence of meteorological determinants. *medRxiv,* DOI: 10.1101/2020.05.26.20113985
4. Ahmadi M, Sharifi A, Dorosti S, Ghoushchi SJ, Ghanbari N (2020). Investigation of effective climatology parameters on COVID-19 outbreak in Iran. *Science of The Total Environment*, 729. DOI: 10.1016/j.scitotenv.2020.138705
5. Ahmed A, Rahman MM (2020). COVID-19 trend in Bangladesh: deviation from epidemiological model and critical analysis of the possible factors. *medRxiv,* DOI: 10.1101/2020.05.31.20118745
6. Ahmed J, Hasnat Jaman M, Saha G, Ghosh P (2020). Effect of temperatures, humidity and population density on the spreading of Covid-19 at 70 cities/provinces. *Preprints,* DOI: 10.20944/preprints202006.0292.v1
7. Alipio M (2020). Do latitude and ozone concentration predict Covid-2019 cases in 34 countries? *SSRN,* DOI: 10.2139/ssrn.3572114
8. Araújo MB, Naimi B (2020). Spread of SARS-CoV-2 Coronavirus likely to be constrained by climate. *MedRxiv*, DOI: 10.1101/2020.03.12.20034728
9. Auler, AC, Cássaro FAM, da Silva VO, Pires LF (2020). Evidence that high temperatures and intermediate relative humidity might favor the spread of COVID-19 in tropical climate: A case study for the most affected Brazilian cities. *Science of The Total Environment*, 729, DOI: 10.1016/j.scitotenv.2020.139090
10. Awasthi R, Nagori A, Singh P, Pal R, Joshi V, Sethi T (2020). Temperature and humidity do not influence global COVID-19 incidence as inferred from causal models. *medRxiv,* DOI: 10.1101/2020.06.29.20142307
11. Ayanshina OA, Adeshakin AO, Afolabi LO, Adeshakin FO, Alli-Balogun GO, Essien-Baidoo S, Wan X (2020). Seasonal variations and immune responses: Any succor for COVID-19 pandemic in Nigeria. *Preprints,* DOI: 10.20944/preprints202006.0030.v1
12. Bäcker A (2020). Slower COVID-19 morbidity and mortality growth at higher solar irradiance and elevation. *SSRN,* DOI: 10.2139/ssrn.3604729.
13. Baker RE, Yang W, Vecchi GA, Metcalf CJE, Grenfell BT (2020). Susceptible supply limits the role of climate in the early SARS-CoV-2 pandemic. *Science*, DOI: 10.1126/science.abc2535
14. Bannister-Tyrrell M, Meyer A, Faverjon C, Cameron A (2020). Preliminary evidence that higher temperatures are associated with lower incidence of COVID-19, for cases reported globally up to 29th February 2020. *medRxiv*, DOI: 10.1101/2020.03.18.20036731
15. Bashir MF, Ma B, Bilal KB, Bashir MA, Tan D, Bashir M (2020). Correlation between climate indicators and COVID-19 pandemic in New York, USA. *Science of the Total Environment*, 728, DOI: 10.1016/j.scitotenv.2020.138835
16. Bellali H, Chtioui N, Chahed M (2020). Factors associated with country-variation in COVID-19 morbidity and mortality worldwide: an observational geographic study. *medRxiv,* DOI: 10.1101/2020.05.27.20114280
17. Bezabih YM, Mequanint A, Alamneh E, Bezabih A, Sabiiti W, Roujeinikova A, Bezabhe WM (2020). Correlation of the global spread of coronavirus disease-19 with atmospheric air temperature. *medRxiv,* DOI: 10.1101/2020.05.27.20115048
18. Bhattacharjee S (2020). Statistical investigation of relationship between spread of coronavirus disease (COVID-19) and environmental factors based on study of four mostly affected places of China and five mostly affected places of Italy. *arXiv,* 2003.11277
19. Briz-Redón Á, Serrano-Aroca Á (2020). A spatio-temporal analysis for exploring the effect of temperature on COVID-19 early evolution in Spain. *Science of The Total Environment*, 728, DOI: 10.1016/j.scitotenv.2020.138811
20. Bu J, Peng DD, Xiao H, Yue Q, Han Y, Lin Y, Hu G, Chen J (2020). Analysis of meteorological conditions and prediction of epidemic trend of 2019-nCoV infection in 2020. *medRxiv*, DOI: 10.1101/2020.02.13.2002271
21. Bukhari Q, Jameel Y (2020). Will coronavirus pandemic diminish by summer? *SSRN*, DOI: 10.2139/ssrn.3556998
22. Cao H, Li B, Gu T, Liu X, Meng K, Zhang L (2020). Associations of ambient air pollutants and meteorological factors with COVID-19 transmission in 31 Chinese provinces: a time-series study. *medRxiv,* DOI: 10.1101/2020.06.24.20138867
23. Carlton T, Cornetet J, Huybers P, Meng KC, Proctor J (2020). Evidence for ultraviolet radiation decreasing COVID-19 growth rates: global estimates and seasonal implications. *SSRN,* DOI: 10.2139/ssrn.3588601
24. Carlton T, Meng KC (2020). Causal empirical estimates suggest COVID-19 transmission rates are highly seasonal. Unpublished, available at: https://github.com/emlan-ucsb/COVID-seasonality
25. Caspi G, Shalit U, Kristensen SL, Aronson D, Caspi L, Rossenberg O, Shina A, Caspi O (2020). Climate effect on COVID-19 spread rate: an online surveillance tool. *medRxiv*, DOI: 10.1101/2020.03.26.20044727
26. Chatziprodromidou I, Apostolou T, Vantarakis A (2020). COVID-19 and environmental factors. A PRISMA-compliant systematic review. *MedRxiv,* DOI: 10.1101/2020.05.10.20069732
27. Chen B, Liang H, Yuan X, Hu Y, Xu M, Zhao Y, Zhang B, Tian F, Zhu X (2020a). Roles of meteorological conditions in COVID-19 transmissions on a worldwide scale. *medRxiv*, DOI: 10.1101/2020.03.16.20037168
28. Chen C, Li X, Meng X, Ma Z, Li W, Dong L (2020b). A retrospective study: meteorological factors and COVID-19. *Research Square,* DOI: 10.21203/rs.3.rs-28151/v1
29. Choi Y-W, Tuel A, Eltahir EAB (2020). An environmental determinant of viral respiratory disease. *medRxiv,* DOI: 10.1101/2020.06.05.20123349
30. Cohen F, Schwartz M, Li S, Lu Y, Jani A (2020). The challenge of using epidemiological case count data: The example of confirmed COVID-19 cases and the weather. *medRxiv,* DOI: 10.1101/2020.05.21.20108803
31. Coro G (2020). A global-scale ecological niche model to predict SARS-CoV-2 coronavirus infection rate. *Ecological Modelling*, 109187.
32. Correa-Araneda F, Ulloa-Yañez A, Núñez D, Boyero L, Tonin AM, Cornejo A, Urbina M, Díaz ME, Figueroa-Muñoz G, Esse C (2020). Environmental determinants of COVID-19 transmission across a wide climatic gradient in Chile. *Research Square,* DOI: 10.21203/rs.3.rs-30393/v1
33. Corripio JG, Raso L (2020). Weather variables impact on COVID019 incidence. *medRxiv,* DOI: 10.1101/2020.06.08.20125377
34. da Silva FL, Gomes MDA, da Silva APL, de Sousa SC, de Souza MFS, da Silva GLP (2020). Correlation between meteorological factors and COVID-19 infection in the Belém Metropolitan region. *medRxiv,* DOI: 10.1101/2020.06.10.20127506
35. Damette O, Goutte S (2020). Weather, pollution and Covid-19 spread: a time-series and Wavelet reassessment. *HAL Science L’Homme et de la Société,* ID: halshs-02629139.
36. Das K, Das Chatterjee N (2020a). Examine the impact of weather and ambient air pollution parameters on daily case of COVID-19 in India. *medRxiv,* DOI: 10.1101/2020.06.08.20125401
37. Das P, Manna S, Basak P (2020). Analyzing the effect of temperature on the outspread of COVID-19 around the globe. *medRxiv,* DOI: 10.1101/2020.05.19.20107433
38. de Gennaro S (2020). The climate divide of Covid-19. *Research Gate (preprint),* available at: https://www.researchgate.net/publication/341611854_The_climate_divide_of_Covid-19
39. Del Rio C, Camacho-Ortiz A (2020). Will environmental changes in temperature affect the course of COVID-19? *The Brazilian Journal of Infectious Diseases*, DOI: 10.1016/j.bjid.2020.04.007
40. Demongeot J, Flet-Berliac Y, Seligmann H (2020). Temperature Decreases Spread Parameters of the New Covid-19 Case Dynamics. *Biology*, 9(5), DOI: 10.3390/biology9050094
41. Dixit A, Vishnoi S, Paul SB (2020). Adding structure to statistics: A study on COVID-19 dynamics in India. *medRxiv,* DOI: 10.1101/2020.05.26.20113522
42. Ficetola GF, Rubolini D (2020). Climate affects global patterns of Covid-19 early outbreak dynamics. *medRxiv,* DOI: 10.1101/2020.03.23.20040501
43. Figueiredo AM, Daponte-Codina A, Figueiredo DCMM, Vianna RPT, de Lima KC, Gil-García yE (2020). Factors associated with the incadence and mortality from COVID-19 in the autonomous communities of Spain. *G Model,* DOI: 10.1016/j.gaceta.2020.05.004
44. Franco R, Galo-Fernandez M (2020a). Environmental temperature does not correlate with COVID-19 spreading and death toll. *OSFPreprints,* DOI: 10.31219/osf.io/5fgrk
45. Franco R, Galo-Fernandez M (2020b). Why temperature cannot correlate with acute COVID-19 spreading and death toll. Data from Spain. *Preprint,* available at: http://galo.es/wp-content/uploads/2020/05/COVID-19-spreading-and-death-1.pdf
46. Gao M, Zhou G, Zhang S, Yung KKL, Guo Y (2020). Non-linear modulation of COVID-19 transmission by climate conditions. *SSRN,* DOI: 10.2139/ssrn.3603090
47. Gunthe SS, Swain B, Patra SS, Amte A (2020). On the global trends and spread of the COVID-19 outbreak: preliminary assessment of the potential relation between location-specific temperature and UV index. *Journal of Public Health*, DOI: 10.1007/s10389-020-01279-y
48. Guo XJ, Zhang H, Zeng YP (2020). Transmissibility of COVID-19 and its association with temperature and humidity. *Europe PMC,* DOI: 10.21203/rs.3.rs-17715/v1
49. Gupta A, Banerjee S, Das S (2020a). Significance of geographical factors to the COVID-19 outbreak in India. *Modelling Earth Systems and Environment,* DOI: 10.1007/s40808-020-00838-2
50. Gupta A, Gharehgozli A (2020). Developing a machine learning framework to determine the spread of COVID-19. *SSRN,* DOI: 10.2139/ssrn.3635211
51. Gupta A, Pradhan B (2020). Impact of daily weather on COVID-19 outbreak in India. *medRxiv,* DOI: 10.1101/2020.06.15.20131490
52. Gupta S, Raghuwanshi GS, Chanda A (2020b). Effect of weather on COVID-19 spread in the US: A prediction model for India in 2020. *Science of the Total Environment, 728*, DOI: 10.1016/j.scitotenv.2020.138860
53. Hasan NA, Siddik S (2020). Possible role of meteorological variables in COVID-19 spread: A case study from a subtropical monsoon country, Bangladesh. *Preprints,* DOI: 10.20944/preprints202006.0347.v1
54. Hirata A, Kodera S, Gomez-Tames J, Rashed EA (2020). Influence of absolute humidity and population density on COVID-19 spread and decay durations: Multi-prefecture study in Japan. *arXiv,* 2006.02197.
55. Huang S, Liu J, Xiong H, Huang J, An H, Dou D (2020). The weather impacts the outbreak of COVID-19 in mainland China. *arXiv,* 2006.1037v1.
56. Iqbal N, Fareed Z, Shahzad F, He X, Shahzad U, Lina M (2020a). Nexus between COVID-19, temperature and exchange rate in Wuhan City: New findings from Partial and Multiple Wavelet Coherence. *Science of The Total Environment*, 729, DOI: 10.1016/j.scitotenv.2020.138916
57. Iqbal MM, Abid I, Hussain S, Shahzad N, Waqas MS, Iqbal MJ (2020b). The effects of regional climatic condition on the spread of COVID-19 at global scale. *Science of the Total Environment,* DOI: 10.1016/j.scitotenv.2020.140101
58. Islam N, Shabnam S, Erzurumluoglu AM (2020). Temperature, humidity, and wind speed are associated with lower Covid-19 incidence. *medRxiv,* DOI: 10.1101/2020.03.27.20045658
59. Jahangiri M, Jahangiri M, Najafgholipour M (2020). The sensitivity and specificity analyses of ambient temperature and population size on the transmission rate of the novel coronavirus (COVID-19) in different provinces in Iran. *Science of the Total Environment*, 728, DOI: 10.1016/j.scitotenv.2020.138872
60. Jamil T, Alam I, Gojobori T, Duarte CM (2020). No evidence for temperature-dependence of the covid-19 epidemic. *medRxiv,* DOI: 10.1101/2020.03.29.20046706
61. Jebril N (2020). Predict the transmission of COVID-19 under the effect of air temperature and relative humidity over the year in Baghdad, Iraq. *SSRN,* DOI: 10.2139/ssrn.3579718
62. Jia J, Ding J, Liu S, Liao G, Li J, Duan B, Wang G, Zhang R (2020). Modeling the control of COVID-19: Impact of policy interventions and meteorological factors. *arXiv:* 2003.02985
63. Jüni P, Rothenbühler M, Bobos P, MMath KET, da Costa BR, Fisman DN, Slutsky AS, Gesink D (2020). Impact of climate and public health interventions on the COVID-19 pandemic: a prospective cohort study. *CMAJ,* 192:566-573.
64. Karimi SM, Majbouri M, White K, Little B, McKinney WP, DuPre N (2020). Spring weather and COVID-19 deaths in the U.S. *medRxiv,* DOI: 10.1101/2020.06.20.20136259
65. Karimuzzaman M, Afroz S, Hossain MM, Rahman A (2020). Forecasting the COVID-19 pandemic with climate variables for top five burdening and three South Asian countries. *medRxiv,* DOI: 10.1101/2020.05.12.20099044
66. Knittel CR, Ozaltun B (2020). What does and does not correlate with COVID-19 death rates? *medRxiv,* DOI: 10.1101/2020.06.09.20126805
67. Kotsiou OS, Kotsios VS, Lampropoulos I, Zidros T, Zarogiannis SG, Gourgoullianis KI (2020). High temperature slows coronavirus disease 2019 transmission rate: A within and among country analysis. *Research Square,* DOI: 10.21203/rs.3.rs-33255/v1
68. Leung NY, Bulterys MA, Bulterys PL (2020). Predictors of COVID-19 incidence, mortality, and epidemic growth rate at the country level. *medRxiv,* DOI: 10.1101/2020.05.15.20101097
69. Lin J, Huang W, Wen M, Ma S, Hua J, Hu H, Li D, Yin S, Qian Y, Chen P, Zhang Q, Yuan N, Sun S (2020). Containing the spread of coronavirus disease 2019 (COVID-19): Meteorological factors and control strategies. *medRxiv,* DOI: 10.1101/2020.05.23.20111468
70. Liu J, Zhou J, Yao J, Zhang X, Li L, Xu X, He X, Wang B, Fu S, Niu T, Yan J, Shi Y, Ren X, Niu J, Zhu W, Li S, Luo B, Zhang K (2020a). Impact of meteorological factors on the COVID-19 transmission: A multi-city study in China. *Science of the Total Environment*, 726, DOI: 10.1016/j.scitotenv.2020.138513
71. Lolli S, Chen Y-C, Wang S-H, Vivone G (2020). Impact of meteorology and air pollution on Covid-19 pandemic transmission in Lombardy region, Nothern Italy. *Research Square,* DOI: 10.21203/rs.3.rs-39280/v1
72. Luo W, Majumder MS, Liu D, Poirier C, Mandl KD, Lipsitch M, Santillana M (2020). The role of absolute humidity on transmission rates of the COVID-19 outbreak. *medRxiv*, DOI: 10.1101/2020.02.12.20022467
73. Magurano F, Baggieri M, Marchi A, Rezza G, Nicoletti L (2020). SARS-CoV-2 infection: the environmental endurance of the virus can be influenced by the increase of temperature. *medRxiv,* DOI: 10.1101/2020.05.30.20099143
74. Mandal CC, Panwar MS (2020). Can the summer temperatures reduce COVID-19 cases? *Public Health,* 185:72-79.
75. Meraj G, Farooq M, Singh SK, Romshoo SA, Nathawat MS, Kanga S (2020). Coronavirus pandemic vs. Temperature in the context of Indian subcontinent – A preliminary statistical analysis. *Research Square,* DOI: 10.21203/rs.3.rs-35809/v1
76. Merow C, Urban MC (2020). Seasonality and uncertainty in COVID-19 growth rates. *medRxiv*, DOI: 10.1101/2020.04.19.20071951
77. Mollalo A, Vahedi B, Rivera KM (2020). GIS-based spatial modelling of COVID-19 incidence rate in the continental United States. *Science of the Total Environment*, 728. DOI: 10.1016/j.scitotenv.2020.138884
78. Munnangi S, Thottempudi SG, Somasamudram VG (2020). Effect of population and weather parameters on the spread of COVID-19 pandemic (A data science approach with German weather dataset). *Academia.edu (Preprint),* available at http://www.academia.edu/download/63500463/Effect_of_Population_and_Weather_Parameters_on_the_spread_of_COVID-19_Pandemic.pdf
79. Muñoz-Cacho P, Hernandez JL, Lopez-Hoyos M, Martínez-Taboada VM (2020). Can climatic factors explain the differences in Covid-19 incidence and severity across the Spanish regions? An ecological study. *Research Square,* DOI: 10.21203/rs.3.rs-29377/v1
80. Neher RA, Dyrdak R, Druelle V, Hodcroft EB, Albert J (2020). Potential impact of seasonal forcing on a SARS-CoV-2 pandemic. *Swiss Medical Weekly*, 150(1112), DOI: 10.4414/smw.2020.20224
81. Neto RAA, Melo GC (2020). Correlation between weather, population size and COVID-19 pandemic: a study of Brazilian capitals. *Journal of Health & Biological Sciences*, 8(1), 1-5.
82. NoghaniBehambari H, Salari M, Noghani F, Tavassoli N (2020). The effect of temperature on Covid-19 confirmed cases: Evidence from US counties. *arXiv,* 2005.12264.
83. Notari A (2020). Temperature dependence of COVID-19 transmission. *arXiv*, 2003.12417.
84. Oliveiros B, Caramelo L, Ferreira NC, Caramelo F (2020). Role of temperature and humidity in the modulation of the doubling time of COVID-19 cases. *medRxiv*, DOI: 10.1101/2020.03.05.20031872
85. Paez A, Lopez FA, Menezes T, Cavalcanti R, Pitta MGDR (2020). A spatio‐temporal analysis of the environmental correlates of COVID‐19 incidence in Spain. *Geographical Analysis*, DOI: 10.1111/gean.12241
86. Passerini G, Mancinelli E, Morichetti M, Virgili S, Rizza U (2020). A preliminary investigation of the statistical correlations between Sars-CoV-2 spread and local meteorology. *International Journal of Environmental Research and Public Health,* DOI: 10.3390/ijerph17114051.
87. Pirouz B, Shaffiee Haghshenas S, Pirouz B, Shaffiee Haghshenas S, Piro P (2020). Development of an Assessment Method for Investigating the Impact of Climate and Urban Parameters in Confirmed Cases of COVID-19: A New Challenge in Sustainable Development. *International Journal of Environmental Research and Public Health*, 17(8):2801
88. Poirier C, Luo W, Majumder MS, Liu D, Mandl KD, Mooring TA, Santillana M (2020). The role of environmental factors on transmission rates of the COVID-19 outbreak: An initial assessment in two spatial scales. *SSRN*, DOI: 10.2139/ssrn.3552677
89. Pramanik M, Chowdhury K, Rana MJ, Bisht P, Pal R, Szabo S, Pal I, Behera B, Liang Q, Padmadas SS, Udmal P (2020). Climatic influence on the magnitude of COVID-19 outbreak: a stochastic model-based global analysis. *medRxiv,* DOI: 10.1101/2020.06.02.20120501.
90. Prata DN, Rodrigues W, Bermejo PH (2020). Temperature significantly changes COVID-19 transmission in (sub) tropical cities of Brazil. *Science of the Total Environment*, 729, DOI: 10.1016/j.scitotenv.2020.138862
91. Qi H, Xiao S, Shi R, Ward MP, Chen Y, Tu W, Su Q, Wang W, Wang X, Zhang Z (2020). COVID-19 transmission in Mainland China is associated with temperature and humidity: A time-series analysis. *Science of the Total Environment,* 728, DOI: 10.1016/j.scitotenv.2020.138778
92. Rahman A, Hossain G, Singha AC, Islam S, Islam A (2020a). A retrospective analysis of influence of environmental/air temperature and relative humidity on SARS-CoV-2 outbreak. *Preprints*, DOI: 10.20944/preprints202003.0325.v1
93. Rahman M, Islam M, Shimanto MH, Ferdous J, Rahman AA-NS, Sagor PS, Chowdhury T (2020b). Temperature extreme may exaggerate the mortality risk of COVID-19 in the low- and middle-income countries: A global analysis. *Preprints,* DOI: 10.20944/preprints202006.0369.v1
94. Rasul A, Balzter H (2020). Relationship between monthly climatic variables and worldwide confirmed COVID-19 cases. *SSRN,* DOI: 10.2139/ssrn.3626108
95. Ren Y, Chen S (2020). Determining the role of air temperature in predicting and controlling COVID-19 risk levels anywhere anytime using multiple modelling analyses. *Preprints,* DOI: 10.20944/preprints2020060373
96. Rios V, Gianmoena L (2020). Is there a link between temperatures and COVID-19 contagions? Evidence from Italy. *medRxiv,* DOI: 10.1101/2020.05.13.20101261
97. Rodrigues SA, Dal Pai A, Grotto RMT, Sarnighausen VCR (2020). Meteorological variables associations and the occurrence of COVID-19 in the City of São Paulo, Brazil. *Revista Ibero­-Americana de Ciências Ambientias,* 11(5).
98. Roy I (2020). Influence of temperature on the global spread of COVID-19. *Preprints,* DOI: 10.20944/preprints202003.0366.v3
99. Sahafizadeh E, Sartoli S (2020). High temperature has no impact on the reproduction number and new cases of COVID-19 in Bushehr, Iran. *medRxiv,* DOI: 10.1101/2020.06.14.20130906
100. Şahin, M (2020). Impact of weather on COVID-19 pandemic in Turkey. *Science of the Total Environment*, 728, DOI: 10.1016/j.scitotenv.2020.138810
101. Sajadi MM, Habibzadeh P, Vintzileous A, Shokouhi S, Miralles-Wilhelm F, Amoroso A (2020). Temperature, Humidity, and Latitude Analysis to Estimate Potential Spread and Seasonality of Coronavirus Disease 2019 (COVID-19). *JAMA Network Open.* 3(6):e2011834-
102. Scafetta N (2020). Distribution of the SARS-CoV-2 Pandemic and its monthly forecast based on seasonal climate parameters. *International Journal of Environmental Research and Public Health,* 17, DOI: 10.3390/ijerph17103493
103. Sethwala A, Akbarally M, Better N, Lefkovits J, Grigg L, Akbarally H (2020). The effect of ambient temperature on worldwide COVID-19 cases and deaths – an epidemiological study. *medRxiv,* DOI: 10.1101/2020.05.15.20102798
104. Shahzad K, Shahzad U, Iqbal N, Shahzad F, Fareed Z (2020). Effects of climatological parameters on the outbreak spread of COVID-19 in highly affected regions of Spain. *Research Square,* DOI: 10.21203/rs.3.rs-30377/v1
105. Shi P, Dong Y, Yan H, Li X, Zhao C, Liu W, He M, Tang S, Xi S (2020). The impact of temperature and absolute humidity on the coronavirus disease 2019 (COVID-19) outbreak-evidence from China. *medRxiv*, DOI: 10.1101/2020.03.22.20038919
106. Singh K, Agarwal A (2020). Impact of weather indicators on the COVID-19 outbreak: A multi-state study in India. *medRxiv,* DOI: 10.1101/2020.06.14.20130666
107. Skutsch M, Dobler C, McCall MBB, Ghilardi A, Salinas M, McCall MK, Sanchez F (2020). The association of UV with rates of COVID-19 transmission and deaths in Mexico: the possible mediating role of vitamin D. *medRxiv,* DOI: 10.1101/2020.05.25.20112805
108. Sobral MFF, Duarte GB, da Penha Sobral AIG, Marinho MLM, de Souza Melo A (2020). Association between climate variables and global transmission of SARS-CoV-2. *Science of The Total Environment*, 729, DOI: 10.1016/j.scitotenv.2020.138997
109. Sobur A, Islam S, Haque E, Rahman AMMT, Islam T, Toniolo A, Rahman T (2020). Temperature and relative humidity are not major contributing factor on the occurrence of COVID-19 pandemic: An observational study in 57 countries (2020-05-08). *medRxiv,* DOI: 10.1101/2020.05.03.20089342
110. Suhaimi NF, Jalaludin J, Latif MT (2020). Demystifying a possible relationship between COVID-19, air quality and meteorological factors: Evidence from Kuala Lumpur, Malaysia. *Aerosol and Air Quality Research,* 20:1520-1529
111. Takagi H, Kuno T, Yokoyama Y, Ueyama H, Matsushiro T, Hari Y, Ando T (2020). Higher temperature, pressure, and ultraviolet are associated with less COVID-19 prevalence – Meta-regression of Japanese prefectural data. *medRxiv,* DOI: 10.1101/2020.05.09.20096321
112. Thangariyal S, Rastogi A, Tomar A, Bhadoria A, Baweja S (2020). *medRxiv,* DOI: 10.1101/2020.06.13.20130138
113. Tobías A, Molina T (2020). Is temperature reducing the transmission of COVID-19? *Environmental Research*, DOI: 10.1016%2Fj.envres.2020.109553
114. Tosepu R, Gunawan J, Effendy DS, Ahmad OAI, Lestari H, Bahar H, Asfian P (2020). Correlation between weather and Covid-19 pandemic in Jakarta, Indonesia. *Science of the Total Environment, 725*, DOI: 10.1016/j.scitotenv.2020.138436
115. Ujiie M, Tsuzuki S, Ohmagari N (2020). Effect of temperature on the infectivity of COVID-19. *International Journal of Infectious Diseases*, DOI: 10.1016/j.ijid.2020.04.068
116. Velásquez RMA, Lara JVM (2020). Gaussian approach for probability and correlation between the number of COVID-19 cases and the air pollution in Lima. *Urban Climate,* DOI: 10.1016/j.uclim.2020.100664
117. Wan X, Chen C, Zhang Z (2020). Early transmission of COVID-19 has an optimal temperature but late transmission decreases in warm climates. *medRxiv,* DOI: 10.1101/2020.05.14.20102459
118. Wang J, Tang K, Feng K, Lv W (2020b). High temperature and high humidity reduce the transmission of COVID-19. *arXiv*: arXiv:2003.05003v3
119. Wang M, Jiang A, Gong L, Luo L, Guo W, Li C (2020a). Temperature Significantly Change COVID-19 Transmission in 429 cities. *medRxiv*. DOI: 10.1101/2020.02
120. Ward MP, Xiao S, Zhang Z (2020). The role of climate during the COVID-19 epidemic in New South Wales, Australia. *Transboundary and Emerging Disease,* DOI: 10.1111/tbed.13631
121. Wen M, Chen L (2020). Impacts of regional climate on the COVID-19 pandemic in 116 countries and territories. *medRxiv,* DOI: 10.1101/2020.06.13.20130013
122. Wen X, Liu C, Wang S, Zhang Y, Zhong R (2020). Relationship between the COVID-19 outbreak and temperature, humidity and solar radiation across China. *SSRN,* DOI: 10.2139/ssrn.3594115
123. Wilson DJ (2020). Weather, social distancing, and the spread of COVID-19. Federal Reserve Bank of San Francisco Working Paper 2020-23. DOI: 10.24148/wp2020-2
124. Wu Y, Jing W, Liu J, Ma Q, Yuan J, Wang Y, Du M, Liu M (2020). Effects of temperature and humidity on the daily new cases and new deaths of COVID-19 in 166 countries. *Science of the Total Environment, 729*, DOI: 10.1016/j.scitotenv.2020.139051
125. Xu H, Yan C, Fu Q, Xiao K, Yu Y, Han D, Wang W, Cheng J (2020a). Possible environmental effects on the spread of COVID-19 in China. *Science of the Total Environment, 731*, DOI: 10.1016/j.scitotenv.2020.139211
126. Xu R, Rahmandad H, Gupta M, DiGennaro C, Ghaffarzadegan N, Amini H, Jalali MS (2020b). The modest impact of weather and air pollution on COVID-19 transmission. *medRxiv,* DOI: 10.1101/2020.05.05.20092627.
127. Yao Y, Pan J, Liu Z, Meng X, Wang W, Kan H, Wang W (2020). No Association of COVID-19 transmission with temperature or UV radiation in Chinese cities. *European Respiratory Journal*, *55*(5), DOI: 10.1183/13993003.00517-2020
128. Zhou J, Qin L, Liu N (2020). Ambient air pollutants, meteorological factors and their interactions affect confirmed cases of COVID-19 in 120 Chinese cities. *medRxiv,* DOI: 10.1101/2020.05.27.20111542
129. Zhu Y, Xie J (2020). Association between ambient temperature and COVID-19 infection in 122 cities from China. *Science of the Total Environment*, DOI: 10.1016/j.scitotenv.2020.138201
130. Zuo ZK, Ullah S, Yan L, Zheng JH, Han CQ, Zhao HY (2020). CNF-based prediction of COVID-19 transmission without considering NPIs. *ESSOAr,* DOI: 10.1002/essoar.1053239
