# Supplementary figures and images for: Winter Is Coming: A Southern Hemisphere Perspective of the Environmental Drivers of SARS-CoV-2 and the Potential Seasonality of COVID-19
Source: Int J Environ Res Public Health. 2020 Aug 5;17(16):5634. doi: 10.3390/ijerph17165634 (PMC7459895; doi:10.3390/ijerph17165634)

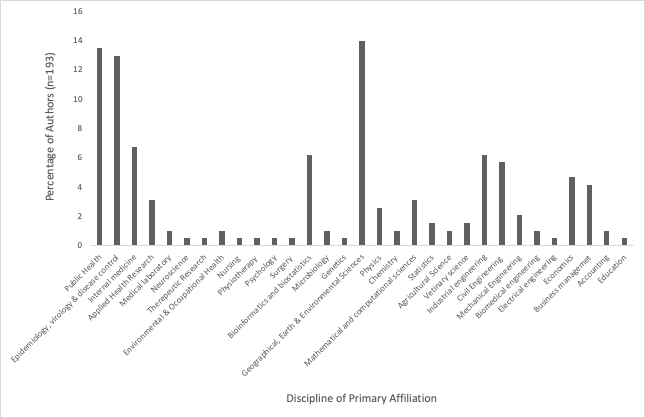


**Figure S1:** Discipline backgrounds of authors whose publications were included in this review.

Supplement: Supplementary file 1 [file ijerph-17-05634-s001.zip › IJERPH Supplement Figure S1 - revision.docx]
